# Alpha-ring Independent Assembly of the 20S Proteasome

**DOI:** 10.1038/srep13130

**Published:** 2015-08-19

**Authors:** Dilrajkaur Panfair, Aishwarya Ramamurthy, Andrew R. Kusmierczyk

**Affiliations:** 1 Department of Biology, Indiana University-Purdue University Indianapolis, 723 West Michigan St. Indianapolis, IN 46202.

## Abstract

Archaeal proteasomes share many features with their eukaryotic counterparts and serve as important models for assembly. Proteasomes are also found in certain bacterial lineages yet their assembly mechanism is thought to be fundamentally different. Here we investigate α-ring formation using recombinant proteasomes from the archaeon *Methanococcus maripaludis*. Through an engineered disulfide cross-linking strategy, we demonstrate that double α-rings are structurally analogous to half-proteasomes and can form independently of single α-rings. More importantly, via targeted mutagenesis, we show that single α-rings are not required for the efficient assembly of 20S proteasomes. Our data support updating the currently held “α-ring first” view of assembly, initially proposed in studies of archaeal proteasomes, and present a way to reconcile the seemingly separate bacterial assembly mechanism with the rest of the proteasome realm. We suggest that a common assembly network underpins the absolutely conserved architecture of proteasomes across all domains of life.

Most intracellular proteins end their existence at the proteasome, a large multifunctional protease complex found in all domains of life. Proteasomes share a common architecture of a central protease capped by one or more regulatory complexes^1^. The regulatory complexes differ in composition, from the hexameric ring-shaped AAA ATPases such as PAN in archaea^2^,^3^ and MpA/ARC in bacteria^4^,^5^ to the ~19 subunit Regulatory Particle of eukaryotes (RP, also called PA700 or 19S proteasome)^6^,^7^. By contrast, the central protease, called the 20S proteasome or core particle (CP), has an absolutely conserved quaternary structure^8^,^9^,^10^,^11^. The CP consists of four stacked heptameric rings. Structurally related subunits, α and β, comprise the outer and inner rings, respectively. Only β subunits are proteolytically active; they are synthesized as proprotein precursors and undergo autocatalytic activation to expose the N-terminal threonine nucleophile^12^,^13^,^14^. Eukaryotic CP rings contain 7 unique α and β subunits, while those of archaea and bacteria usually consist of one or two types of subunit each. Although ubiquitous in archaea and eukaryotes, only a small subset of bacteria possess 20S proteasomes, possibly owing to a horizontal gene transfer from archaea^15^.

Assembly of the proteasome, and of the 20S CP in particular, has garnered considerable attention (recently reviewed in^16^,^17^,^18^). The general consensus posits that α subunits form rings first which act as a platform for the subsequent entry of β subunits^19^,^20^. Incorporation of the β subunits leads to the formation of a double-ring structure, the half-proteasome, which quickly dimerizes to form the 20S CP. A cadre of dedicated chaperones assists in CP assembly in eukaryotes^21^,^22^,^23^,^24^,^25^,^26^,^27^ and a subset of these chaperones may be conserved in archaea^28^. Despite differences in complexity, the assembly of archaeal and eukaryotic CP shares the same mechanism. Consequently, archaea have served as an important model for eukaryotic CP assembly^19^,^29^,^30^. By contrast, the bacterial CP assembles via a different mechanism involving the formation of αβ heterodimers and their subsequent assembly into half-proteasomes^31^,^32^,^33^.

Since early events in CP assembly, including those leading to the formation of α-rings, are not completely understood, we wished to explore them in more detail using recombinantly produced archaeal α subunits as a model. We find that the currently held α-ring first view of CP assembly should be updated to include an alternate, parallel assembly pathway highly reminiscent of bacterial CP assembly. Our findings demonstrate that the common CP architecture across all domains of life is underpinned by common mechanisms of assembly, further underscoring the shared evolutionary origin of this important complex.

## Results

### Archaeal α-rings

**Figure 1:**
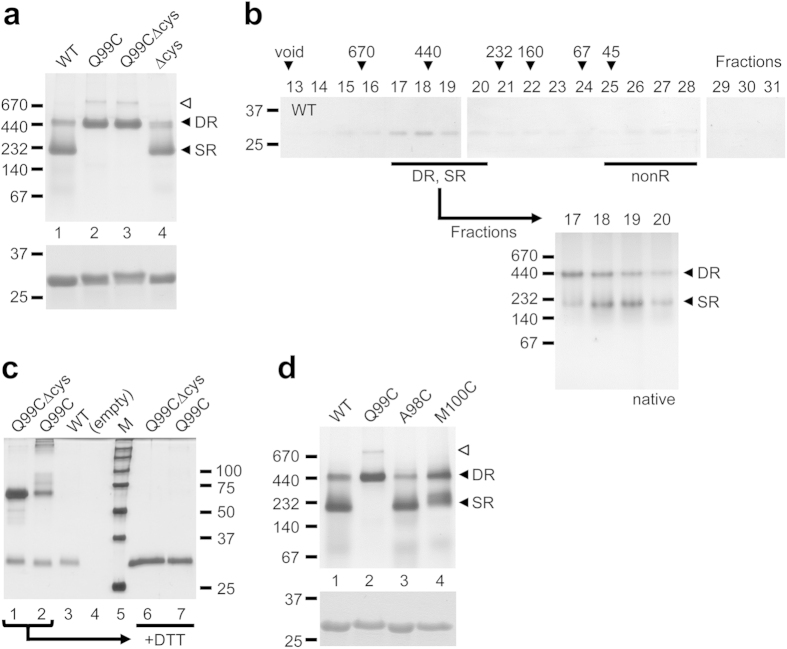
Structural similarity between double α-rings and half-proteasomes. (**a**) Recombinant wild-type (WT) and mutant archaeal α-his subunits (20 μg) were analyzed by nondenaturing 4–15% gradient gel (top panel). Equal protein loading was verified by 12% SDS-PAGE (bottom panel). Proteins visualized by GelCode blue. Black arrowheads denote double α-ring (DR) and single α-ring (SR) species. White arrowhead denotes a gel-induced higher order species. The position of several molecular size standards (in kDa) is indicated in each panel. (**b**) Wild-type α-his protein (780 μg) subjected to size exclusion chromatography on a Sephacryl S-300 column. Indicated fractions were analyzed by three 12% SDS-PAGE gels stained with GelCode blue (top panels). Black arrowheads indicate the column void volume and the elution peaks of molecular size standards (in kDa). Aliquots from the major peak (fractions 17–20) were analyzed on a nondenaturing 5–10% gradient gel stained with silver (bottom panel, “native”). Black arrowheads denote SR and DR species. The position of several molecular size standards (in kDa) is indicated. (**c**) The Q99C mutation results in cross-linked α-rings. Bands corresponding to several DR species were excised from the native gel in (**a**) Proteins within the bands were eluted, analyzed by 12% SDS-PAGE under non-reducing (lanes 1–3), or reducing (lanes 6, 7), conditions, and visualized by silver staining. *M*, molecular size standards (size in kDa indicated at right). (**d**) Q99C is ideally placed to enable cross-linking of α-rings. Recombinant wild-type (WT) and mutant archaeal α-his subunits were purified and analyzed as described in (**a**).

Recombinant archaeal α subunits form single^29^ or double^19^ rings. To investigate early events of α subunit assembly, we expressed C-terminally hexahistidine-tagged (his-tagged) α subunits (α-his) from the archaeaon *Methanococcus maripaludis* S2 in *Escherichia coli*. The α-his protein was purified by immobilized-cobalt affinity resin (ICAR) and analyzed by native PAGE. Two main bands were observed: a prominent lower band near the 232 kDa size standard and a weaker upper band near the 440 kDa size standard (Fig. 1a, lane 1). Size exclusion chromatography confirmed that these two bands represented distinct species; the two elution peaks overlapped in fractions 17–20 (Fig. 1b). A smaller third peak was observed in fractions 25–28 most likely representing free α-his subunits (expected *M*_*r*_ 29.5 kDa); we referred to it as “non-ring” (nonR) to account for the possibility that some dimers might be present. The lower and upper bands on native PAGE were tentatively assigned to be single α-rings (SR; expected *M*_*r*_ 206 kDa) and double α-rings (DR; expected *M*_*r*_ 413 kDa), respectively.

Some eukaryotic α subunits also assemble into DR when expressed in bacteria^34^,^35^. The significance of DR formation is not known and no high resolution data describes their structure. Cryoelectron microscopy (cryoEM) analysis reveals that the two α-rings are offset by ~25° relative to each other^19^. An identical offset exists between α- and β-rings within each half of the CP, contributing to the saw-tooth interdigitation of the subunits^10^ mediated mainly by contact between respective H1 helices (Supplementary Fig. S1). Since α and β subunits are structurally related^10^, one can hypothesize that a DR and an αβ ring pair (i.e. a half-proteasome) exhibit a similar quaternary structure. To test this, we adopted a cross-linking strategy: if DR and half-proteasomes are structurally analogous, it should be possible to cross-link two α subunits with a suitably placed cysteine residue in the H1 helix.

A glutamine at position 99 of the *M. maripaludis* α subunit may be well positioned for an engineered disulfide cross-link (Supplementary Fig. S1). We generated three α subunit mutants by site directed mutagenesis: a mutant with no endogenous cysteines (Δcys); a mutant containing an engineered H1 helix cysteine in addition to the three endogenous cysteines (Q99C); and a mutant containing the H1 helix cysteine but with no endogenous cysteines (Q99CΔcys). We expressed the α-his subunits in *E. coli*, purified them by ICAR, and analyzed them by native PAGE (Fig. 1a, lanes 2–4). Unlike wild-type α subunits, the Q99C and the Q99CΔcys mutants exhibited no SR band but a prominent DR band instead. An even slower-migrating band (white arrowhead) was a gel-induced higher order species (Supplementary Fig. S2). The transition to DR as the major species was absent in the Δcys mutant.

These results were consistent with Q99C-dependent cross-linking of α subunits locking two α-rings together, causing SR to convert to DR. To confirm this, we excised the DR bands from the wild-type, Q99C, and Q99CΔcys samples, eluted the proteins within, and subjected them to SDS-PAGE under non-reducing conditions (Fig. 1c, lanes 1–3). The wild-type sample exhibited only a band at ~30 kDa, corresponding to an α subunit monomer. The Q99C and the Q99CΔcys samples exhibited the monomer band and a new ~60 kDa band, consistent with an α subunit dimer, which disappeared under reducing conditions (Fig. 1c, lanes 6, 7). The lack of endogenous cysteines in the Q99CΔcys mutant means that only the desired cross-links are observed, consistent with the higher intensity of the ~60 kDa band in the Q99CΔcys sample versus the Q99C sample. The cross-linking efficiency was not 100% in either mutant (Fig. 1c), yet the SR to DR shift on native PAGE was complete in both (Fig. 1a). This is explained by only one cross-linked pair of α subunits being needed to lock the two α-rings in a DR. Placing the cross-linkable cysteine even one residue away from position 99 eliminated (A98C), or greatly reduced (M100C), the transition from SR to DR (Fig. 1d). We conclude that the Q99C mutation can cross-link two α subunits in opposite rings together, effectively locking the DR, because the α rings interact via precisely aligned H1 helices. This is strong evidence that our tentative assignment of the lower (SR) and upper (DR) bands on native PAGE was correct and, more importantly, supports the hypothesis that DR are structurally analogous to half-proteasomes.

### Charged residues and α-ring assembly

In the current view of CP assembly, α subunits assemble into single rings (SR) first. Double rings (DR) presumably arise from preformed SR and exist in equilibrium with them; our cross-linking results are consistent with this view. Interactions between H0 helices, present in α but not β subunits, help stabilize the formation of α-rings^19^. We used site directed mutagenesis to investigate what other factors influence ring stability. Based on available structures^10^,^29^, we targeted highly conserved charged residues at α subunit interfaces within the same ring (Supplementary Fig. S3). Those that were close to highly conserved residues of opposite charge on an adjacent subunit might form stabilizing salt bridges; if so, mutating them would destabilize α-rings, interfering with their formation. We expressed two mutant α-his subunits in *E. coli* (K59E and R88D), purified them by ICAR, and analyzed them by native PAGE.

**Figure 2:**
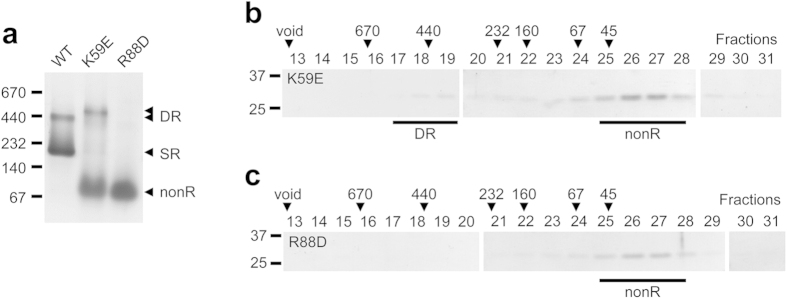
Conserved charged residues at α–α subunit interface contribute to α-ring stability. Recombinant wild-type (WT) and mutant archaeal α-his subunits were purified by immobilized cobalt affinity resin (ICAR) and 10 μg of protein from each sample eluate was analyzed on a nondenaturing 4–15% gradient gel stained with GelCode blue (**a**). Double α-ring (DR), single α-ring (SR), and non-ring (nonR) species are denoted with black arrowheads; nonR denotes α subunits that have not assembled into any ring and consist mostly of free α subunits. The position of several molecular size standards (in kDa) is indicated. (**b,c**) The purified mutant proteins (780 μg) were subjected to size exclusion chromatography on a Sephacryl S-300 column and 3 ml fractions were collected. Aliquots (50 μl) of the indicated fractions were analyzed by three 12% SDS-PAGE gels and stained with GelCode blue. Black lines delineate the position of the DR and nonR peaks. The locations of the column void volume and the elution peaks of the indicated molecular size standards (in kDa) are indicated with black arrowheads.

In both mutants, the SR band was replaced with a much faster migrating species, consistent with these two mutations having a destabilizing effect on α-ring formation (Fig. 2a). This faster migrating species most likely represents free α subunits but, as above, we referred to it as nonR to allow for the possibility of some dimers. For the R88D mutant, the nonR species was the only readily-observed species, arguing that this mutation had a profound effect on ring formation. By contrast, the K59E mutant exhibited a weak band migrating near the DR of the wild-type. We surmised that this was a DR whose mobility on native PAGE was slightly different because the mutation affected the mass-to-charge ratio of the protein. Size exclusion chromatography verified the native PAGE results (Fig. 2b,c and Supplementary Fig. S4). The K59E mutant protein exhibited two peaks, a major peak in fractions 25-28 (nonR) and a minor peak in fractions 17–19 (DR). The R88D mutant protein exhibited only the major peak in fractions 25–28 (nonR). We conclude that perturbing conserved charged residues at the intra-ring α subunit interface interferes with the assembly of SR, but not necessarily DR, and that the R88 residue has a much bigger effect on ring stability.

### α-ring independent 20S assembly

**Figure 3:**
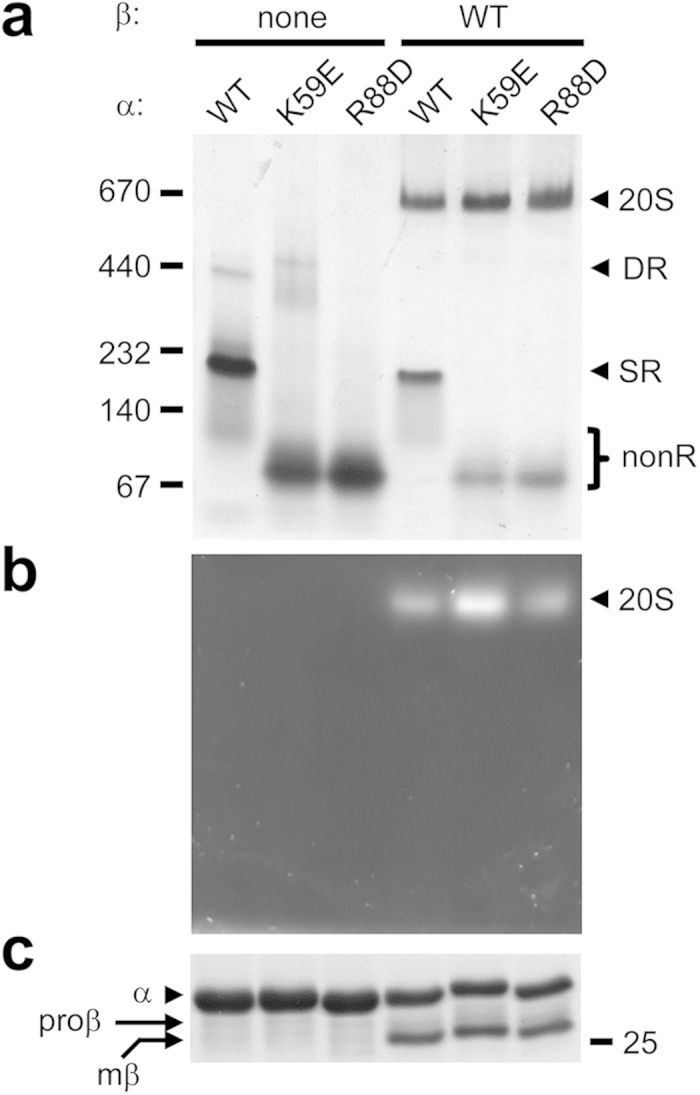
Mutant α subunits form functional 20S proteasomes. (**a,b**) Wild-type (WT) and mutant archaeal α-his subunits were expressed in *E. coli* either individually, or coexpressed with wild-type archaeal β subunits. The recombinant proteins were purified by immobilized cobalt affinity resin (ICAR) and 10 μg of protein from each sample eluate was electrophoresed on a nondenaturing 5–10% gradient gel. Immediately prior to GelCode staining (**a**), the polyacrylamide gel was overlaid with buffer solution containing the fluorogenic peptide substrate Suc-LLVY-AMC to detect peptidase activity (**b**). Black arrowheads denote the positions of assembled 20S core particle (20S), double α-ring (DR) and single α-ring (SR). The position of α subunit species that do not assemble into any ring (nonR), and are mostly free α subunits, is shown with a bracket. The migration of several molecular size standards (in kDa) is indicated. (**c**) Aliquots of the ICAR-purified proteins from (**a**) were also analyzed by 12% SDS-PAGE stained with GelCode blue. Migration of the 25-kDa molecular size standard is indicated.

The SR is considered to be an obligatory assembly intermediate in archaea and eukaryotes while a DR is thought to be an assembly-incompetent complex^22^. Since our two mutants appeared to unable to form any detectable SR (K59E did form some DR), we expected them to be incapable of proteasome formation. To determine if this was the case, we coexpressed *M. maripaludis* α-his and β subunits in bacteria^23^, purified them by ICAR and analyzed them by native PAGE (Fig. 3).

Coexpression of wild-type α and β subunits resulted in a prominent species near the 670 kDa size standard (Fig. 3a). We’ve shown this to be functional 20S^28^ and it exhibited peptidase activity in an in-gel assay (Fig. 3b). Some SR, and a small amount of nonR, was also observed in the wild-type sample but no DR band was present. Unexpectedly, coexpression of both K59E and R88D mutants with wild-type β subunits also resulted in catalytically active bands near the 670 kDa size standard; small amounts of nonR species were observed in each mutant as well. SDS-PAGE analysis revealed both α and primarily mature β subunits in all 3 proteolytically active samples (Fig. 3c). We conclude that the mutant α subunits formed functional proteasomes.

**Figure 4:**
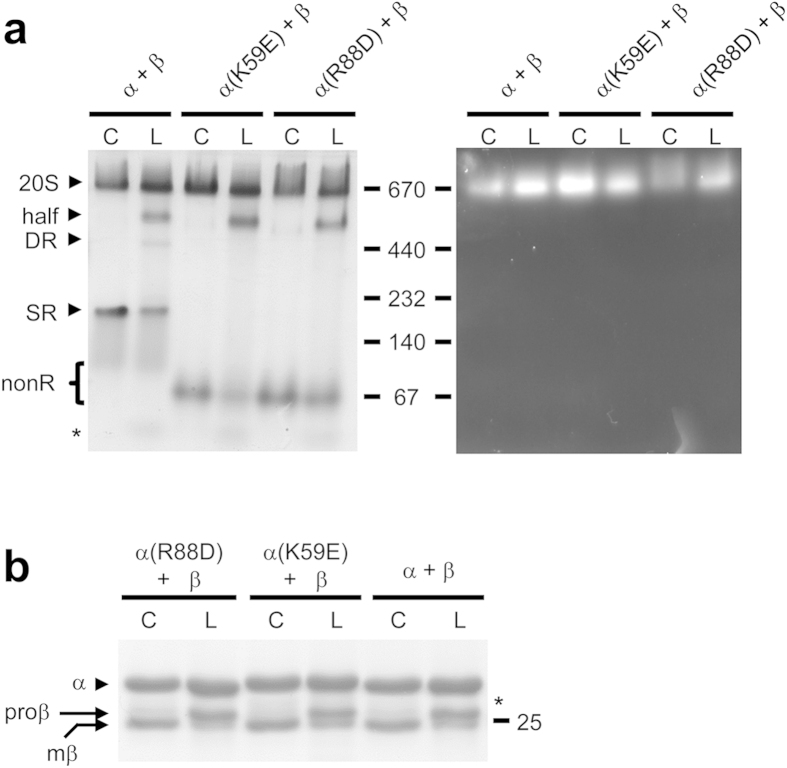
Proteasome assembly assayed by coexpression and lysate mixing. (**a**) For coexpression (C), wild-type (WT) or mutant α-his subunits were coexpressed with wild-type β subunits in *E. coli* and the proteins purified by immobilized cobalt affinity resin (ICAR). For lysate mixing (L), proteasome assembly was initiated by mixing equal volumes of lysates from cells separately expressing the indicated α-his and β subunits, and proteins were purified by ICAR. Purified proteins (10 μg) from each sample eluate were electrophoresed on a nondenaturing 5–10% gradient gel. Immediately prior to GelCode staining (left panel), the gel was overlaid with buffer solution containing the fluorogenic peptide substrate Suc-LLVY-AMC to detect peptidase activity (right panel). Black arrowheads denote the assembled 20S core particle (20S), putative half-proteasome (half), double α-ring (DR) and single α-ring (SR). The position of α subunit species that do not assemble into any ring (nonR), and are mostly free α subunits, is shown with a bracket. The migration of several molecular size standards (in kDa) is indicated. (**b**) Decreased processing of β subunit propeptides during lysate mixing. Aliquots of the ICAR-purified proteins from (**a**) were analyzed by 12% SDS-PAGE stained with GelCode blue. Black arrowhead denotes migration of α-his subunit and arrows indicate position of fully mature (mβ) and immature (proβ) β subunits. The migration of the 25-kDa molecular size standard is shown. Asterisk denotes the migration of a truncated α-his subunit resulting from non-specific proteolysis post lysis; its migration is also apparent on native PAGE in (**a**).

Next, we carried out an important control. Protein assembly is cooperative and strongly concentration dependent^36^. Mutant α subunits appeared incapable of forming SR (Figs 1 and 3), but this conclusion is based on *in vitro* experiments where the purified protein is at much lower concentrations. By contrast, excluded volume effects inside bacteria result in much higher effective protein concentrations^37^. Therefore, one cannot rule out that these higher concentrations during coexpression could promote just enough SR assembly from mutant α subunits to allow β subunits to bind and form CP. To overcome this uncertainty, we expressed α-his and untagged β subunits separately in bacteria and performed lysate mixing prior to purification by ICAR. Since the mutant α subunits do not appear to form SR under the decreased protein concentrations post-lysis (Fig. 2), this eliminated the concentration concerns. The purified proteins were analyzed by native PAGE and, in all cases, functional proteasomes were formed (Fig. 4a). We conclude that formation of SR is not required for assembly of functional proteasomes.

Interestingly, lysate mixing produced a number of changes. First, the DR species reappeared in the wild-type sample. Second, while the coexpressed samples contained mostly fully mature β subunits (mβ), the lysate mixing samples all contained higher levels of immature (proβ) β subunits (Fig. 4b). This argued for much less efficient assembly during lysate mixing relative to coexpression likely because the lower protein concentrations in lysates result in decreased assembly rates. Third, a prominent new band migrating between the 670 and 440 kDa size standards appeared in all three samples; it was slightly more abundant in the two mutants. This band was already faintly present in the mutant samples during coexpression, but it was more prominent during lysate mixing. We tentatively assigned it to be a half-proteasome (Fig. 4a, half) since excision of this band, and elution of the proteins within, revealed comparable levels of α and β subunits (not shown) yet it had no peptidase activity in the in-gel assay (Fig. 4a, right panel).

### Bacterial-like assembly features

Formation of archaeal proteasomes independently of SR could be explained if α and β subunits combined directly to form half-proteasomes. This would imply that archaeal CP assembly can proceed along a pathway similar to bacterial CP assembly^38^. That mutant α subunits can form half-proteasomes without forming SR or DR was suggested by lysate mixing experiments (Fig. 4a, half). We sought data to confirm the identity of this putative half-proteasome and demonstrate that it is an on-pathway intermediate. In the archaeal CP, a highly conserved β subunit arginine (R166 in *M. maripaludis*) in one β ring is well positioned to form stabilizing salt bridges with conserved acidic residues on the opposing β ring (Supplementary Fig. S3 and^10^). We mutated this residue to a tryptophan (R166W), reasoning that this should disrupt half-proteasome dimerization and hence CP assembly. Consequently, levels of the half-proteasome precursor should accumulate while levels of the 20S CP product should decrease, consistent with a precursor-product relationship. Lysates expressing wild-type or mutant α-his subunits were mixed with lysates expressing full length wild-type or mutant β subunits. The mixtures were purified by ICAR and the purified proteins analyzed by native PAGE and in-gel substrate overlay assay.

**Figure 5:**
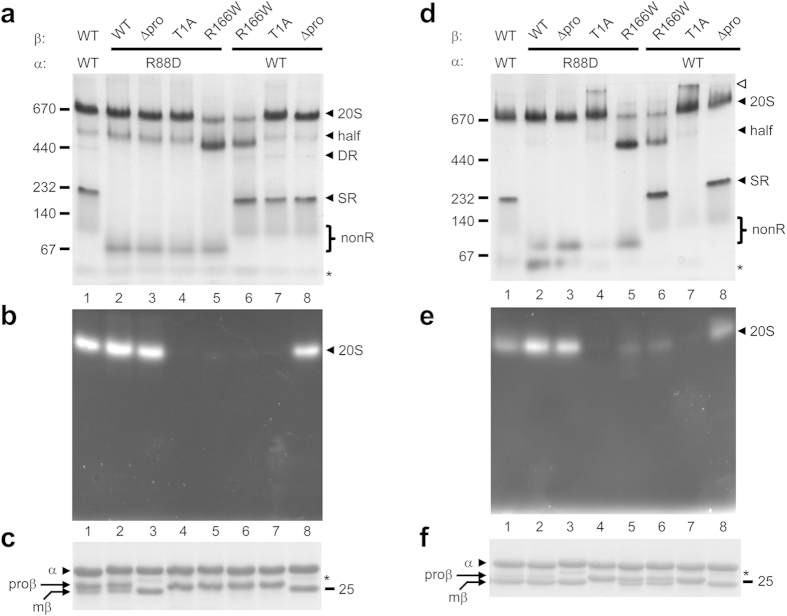
Bacterial-like features of archaeal 20S proteasome assembly. (**a–c**) Lysate mixing. Proteasome assembly was initiated by mixing equal volumes of lysates from *E. coli* cells separately expressing the indicated wild-type (WT) or mutant α-his and b subunits. Proteins were purified by immobilized cobalt affinity resin (ICAR). (**d–f**) Coexpression. Wild-type (WT) or mutant α-his subunits were coexpressed with wild-type or mutant β subunits in *E. coli* and the proteins were purified by ICAR. Purified proteins (10 μg) from each sample eluate were electrophoresed on a nondenaturing 5–10% gradient gel. Immediately prior to staining with GelCode blue (**a,d**) the native gels were overlaid with buffer solution containing the fluorogenic peptide substrate Suc-LLVY-AMC to detect peptidase activity (**b,e**). Black arrowheads denote the assembled 20S core particle (20S), half-proteasome (half), double α-ring (DR) and single α-ring (SR). The position of α subunit species that do not assemble into any ring (nonR), and are mostly free α subunits, is shown with a bracket. White arrowhead denotes a gel-induced higher order species. The migration of several molecular size standards (in kDa) is indicated. (**c,f**) Aliquots of the ICAR-purified proteins from (**a**,**d**) were also analyzed by 12% SDS-PAGE stained with GelCode blue. Black arrowhead denotes migration of α-his subunit and arrows indicate position of fully mature (mβ) and immature (proβ) β subunits. The migration of the 25-kDa molecular size standard is shown. Asterisk denotes a truncated α-his subunit resulting from non-specific proteolysis post lysis; its migration is also apparent on native PAGE in (**a**,**d**).

As before, mixing wild-type α and wild-type β subunits resulted in functional proteasomes; DR, SR, nonR and half species were also present (Fig. 5a,b, lane 1). Mixing wild-type α with mutant β (R166W) subunits resulted in the same banding pattern except the 20S species was greatly reduced and the half species was increased (Fig. 5a, lanes 1 versus 6). The change in the relative intensities of the 20S and half species implies a precursor-product relationship for these two bands. We conclude that the R166W mutation disrupts assembly at the half-proteasome stage and that our tentative assignment of the half species as a half-proteasome was correct.

When α (R88D) was combined with wild-type β subunits, functional proteasomes were again observed (Fig. 5a, lane 2). NonR and half species were also present and the half species was slightly more abundant in the α (R88D) sample than in the wild-type α sample (Fig. 5a, lane 2 versus 1). Finally, when α (R88D) was combined with β (R166W), we saw the same decrease in the 20S species and the same increase in the half species (Fig. 5a, lane 2 versus lane 5). We conclude that the same precursor-product relationship was being observed and that the half species in the α (R88D) mutant samples was also an on-pathway half proteasome.

We did not observe peptidase activity for any sample employing the R166W mutant (Fig. 5b) and this correlated with a lack of fully mature β subunits (Fig. 5c). This is also consistent with the R166W mutation disrupting assembly at the half-proteasome stage, thereby reducing the likelihood of assembly of mature 20S. The slight migration differences of the half-proteasome bands between the various samples (Fig. 5a, lanes 1, 2, 5, 6) was attributed to both mutations (R88D and R166W) being capable of altering the mass-to-charge ratios of this complex relative to wild-type. As might be expected, the degree of accumulation of the half-proteasome was greatest in the double mutant and least in the wild-type sample. We summarize the degree of half-proteasome accumulation in the various mutants as: [α (R88D) β (R166W)] > [α β (R166W)] > [α (R88D) β] > [α β].

We repeated the entire R166W analysis described above (Fig. 5a–c) using subunit coexpression, as opposed to lysate mixing, and obtained essentially identical results (Fig. 5d–f). The major difference was that coexpression, but not lysate mixing, resulted in some 20S activity in the R166W samples (compare corresponding lanes 5 and 6). This was likely due to more efficient maturation during coexpression, evidenced by increased levels of the mature β (mβ) subunit (Fig. 5c,f).

Prior to this study, one aspect of assembly where archaeal and bacterial CP were similar was in the role of the β subunit propeptide: neither required it^19^,^39^ though it greatly improved assembly efficiency in some bacteria^31^. We sought to determine the role of the β subunit propeptide when archaeal α subunits were incapable of forming rings. We employed β subunit mutants lacking the propeptide (Δpro), and ones incapable of cleaving their propeptide due to an active site mutation (T1A)^28^. Functional proteasomes were obtained in both lysate mixing (Fig. 5a–c) and coexpression (Fig. 5d–f) experiments employing β (Δpro), indicating that the propeptide is not required for assembly. Fully assembled, albeit inactive, 20S species were also obtained in both lysate mixing and coexpression experiments employing β (T1A), indicating that a permanently present propeptide does not prevent assembly. We conclude that even when α subunits cannot form rings, archaeal and bacterial CP assembly remain similar with regards to the role of the β subunit propeptide (see Supplementary Note). Two minor differences between lysate mixing and coexpression results, which do not affect this conclusion, are noted in the text that accompanies Supplementary Figure S5. This supplementary figure also demonstrates that results obtained with the α (K59E) mutant were identical to those described for α (R88D) in Fig. 5.

### Assembly-competent species

**Figure 6:**
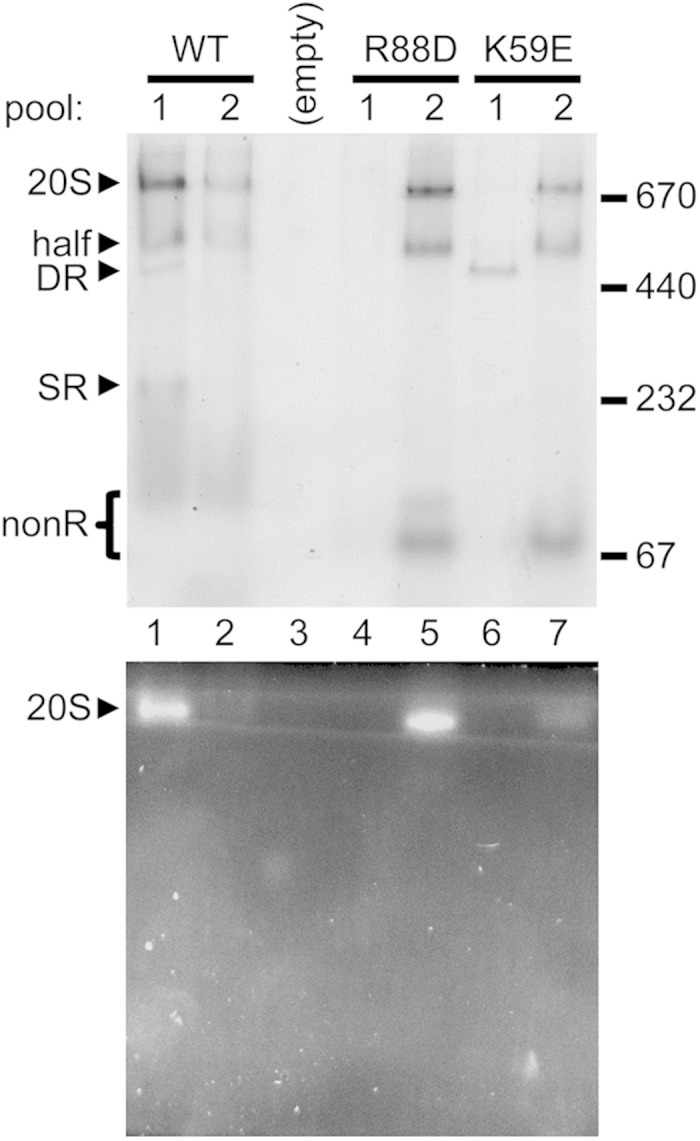
Ring independent assembly of archaeal 20S proteasomes. Recombinant wild-type (WT) and mutant archaeal α-his subunits were purified by immobilized cobalt affinity resin (ICAR). The purified proteins (780 μg) were fractionated by size exclusion chromatography on a Sephacryl S-300 column exactly as described in Figs 1b and 2b,c. For each of the three α-his samples, fractions 17–19 were combined and concentrated (pool 1), and fractions 25–28 were combined and concentrated (pool 2). The three pool 1 and three pool 2 samples were mixed with equal volumes of lysate from *E. coli* expressing wild-type archaeal β subunits. The proteins were repurified by ICAR and equal volumes of each eluate were electrophoresed on a nondenaturing 5–10% gradient gel. Immediately prior to GelCode staining (top panel), the polyacrylamide gel was overlaid with buffer solution containing the fluorogenic peptide substrate Suc-LLVY-AMC to detect peptidase activity (bottom panel). Black arrowheads denote the positions of assembled 20S core particle (20S), half-proteasome (half), double α-ring (DR) and single α-ring (SR). The position of α subunit species that do not assemble into any ring (nonR), and are mostly free α subunits, is shown with a bracket. The migration of several molecular size standards (in kDa) is indicated.

Our data suggest that archaeal α subunits can form proteasomes along an SR-independent pathway, reminiscent of bacterial 20S assembly. We needed to show that the free/unassembled α subunits served as the starting point for this alternative pathway. To this end, we purified wild-type and mutant α-his subunits by ICAR and fractionated them by size exclusion chromatography as before (Figs 1b and 2b). We combined fractions 17–19 into pool 1, corresponding to “ringed species”. As seen in Fig. 1b, the sizing column cannot cleanly separate SR from DR, hence the “ringed species” pool from wild-type subunits contains both SR and DR. We combined fractions 25–28 into pool 2, corresponding to nonR species (mostly free α subunits). The pooled samples were concentrated, mixed with equal volumes of bacterial lysates containing untagged wild-type β subunits, and repurified by ICAR. The repurified samples were analyzed by native PAGE (Fig. 6 and Supplementary Fig. S6).

In the wild-type sample, pool 1 contained the expected SR and DR bands and gave rise to functional CP (Fig. 6, lane 1). Pool 2 also gave rise to functional CP (Fig. 6, lane 2), consistent with the idea that nonR species (mostly free α subunits) can serve as starting material for assembly. There was more CP formed from pool 1 because wild-type α subunits exist primarily as SR and DR (Fig. 1) so this pool contained more α subunits to begin with. The small amount of DR in pool 2 likely formed from free α subunits during sample concentration. The α (K59E) subunits can also form some DR (Fig. 2 and Supplementary Fig. S4) and pool 1 from the α (K59E) mutant sample exhibited a DR band. However, there was very little assembled CP generated from this pool (Fig. 6, lane 6) suggesting that DR is a poor substrate for CP formation. DR have been proposed to be dead-end complexes^22^. The barely-perceptible amount of 20S species formed from this pool could be due to some DR dissociating into assembly competent nonR. A barely-perceptible amount of 20S species was also observed from pool 1 of the α (R88D) mutant (lane 4); a likely reason for this is presented in Supplementary Figure S7.

Unlike wild-type α subunits, both mutant α subunits existed primarily as nonR species (Figs 2 and 3). When these nonR species were used as the starting material for assembly (i.e. pool 2), functional proteasomes formed readily (Fig. 6, lanes 5 and 7). This strongly argues that free α subunits can serve as starting material for SR-independent assembly of CP. Interestingly, all the pools which readily gave rise to functional CP also gave rise to the half-proteasome (Fig. 6, lanes 1, 2, 5, 7). This was consistent with results showing that the half-proteasome is an on-pathway intermediate in both SR-dependent and SR-independent pathways (Fig. 5).

Bacterial 20S proteasomes most likely assemble via αβ heterodimers^31^,^32^,^33^. We wished to determine if the SR-independent assembly of archaeal 20S proteasomes also involved the formation of αβ heterodimers. Time course experiments based on the mixing of separately purified α-his and β-his subunits demonstrated that assembly was rapid (Supplementary Fig. S8). We did not observe any novel bands on nondenaturing gels that would be consistent with αβ heterodimer formation. This could be due to αβ heterodimers being a transient species, which assembles quickly into half-proteasomes, or to αβ heterodimers not being stable enough to survive electrophoresis, or both. As an alternate approach, lysates expressing wild-type or mutant α-his subunits were mixed with lysates expressing untagged full length wild-type or mutant β subunits (see below). The mixtures were purified by ICAR and the purified proteins fractionated by size exclusion chromatography (Supplementary Fig. S9).

When wild-type α-his subunits were mixed with wild-type untagged β subunits, we observed a prominent peak of α and β subunits in fractions 15–18. This peak corresponded to assembled proteasomes and half-proteasomes. The excess of α-his subunits over β subunits in these fractions was due to the presence of DR and some SR, since the Sephacryl S-300 column cannot reliably separate these species (Fig. 1b and not shown). Some free β subunits that may have dissociated during chromatography eluted in fractions 32–34, consistent with the elution position of purified β-his subunits (Supplementary Fig. S8). We also found a small amount of β subunits coeluting with α-his subunits in fractions 25–30. This region contains the nonR α subunit species (Fig. 1b) and is consistent with where an αβ heterodimer (predicted *M*_*r*_, 53.1 kDa) might be expected to elute. When mutant α-his subunits (K59E) were mixed with wild-type untagged β subunits, we again observed a peak in fractions 15–18 corresponding to assembled proteasomes. Here, the levels of α-his and β subunits were approximately equal because the K59E mutant forms very little DR (and no SR). Interestingly, we now observed more β subunits coeluting with the mutant α-his in fractions 25–30 (Supplementary Fig. S9). The K59E mutation generates more nonR species (Fig. 2c). Hence, increased levels of β-subunits in these fractions could be due to more αβ heterodimer formation from the free α subunits in the nonR species, because the SR-dependent assembly pathway is not available to the K59E mutant. Finally, we repeated the analysis with the α-his (K59E) mutant, but employed a β subunit mutant (K29E) that is expected to weaken β–β subunit interactions within a β ring (Supplementary Fig. S9). This β mutant should impair the SR-independent assembly pathway, which is the only assembly pathway operating in the α (K59E) mutant. If the SR-independent pathway involves the formation of αβ heterodimers, we should observe even more β subunits in fractions 25–30 due to the accumulation of these precursors. This is exactly what was observed (Supplementary Fig. S9). Taken together, our results are consistent with the existence of archaeal αβ heterodimers. However, we cannot exclude the possibility of heterotrimers (α_2_β or αβ_2_) given the resolving capacity of the size exclusion column (see also Supplementary Note).

## Discussion

**Figure 7:**
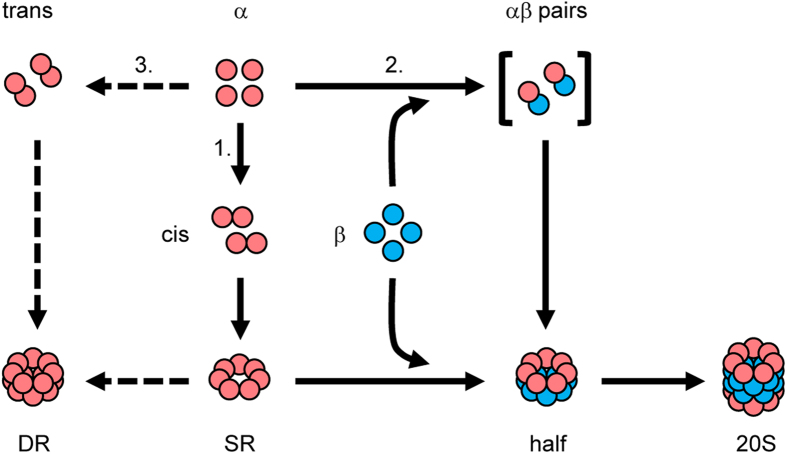
Assembly network for the archaeal 20S proteasome. Three assembly pathways are available to α subunits. The α subunits can interact with each other *in cis* (pathway 1) leading to the formation of an α-ring (SR). The SR acts as a template for β subunit entry until a half-proteasome (half) is formed, which dimerizes to give rise to the core particle (20S). This pathway is followed by archaeal and eukaryotic α subunits. The α subunits can interact with β subunits to form the half-proteasome directly (pathway 2) and independently of SR. Here, the bracket denotes αβ heterodimers as the most likely precursor to half-proteasomes. Pathway 2 is highly reminiscent of bacterial 20S assembly. It is not known if eukaryotic α subunits can follow an SR-independent route. The α subunits can interact with each other *in trans* mediated by contacts between H1 helices (pathway 3) in a manner that would be entirely analogous to the formation of αβ heterodimers. This leads to the formation of a double α-ring (DR) that is structurally analogous to a half-proteasome. This pathway can be followed by archaeal and eukaryotic α subunits. DR can also form directly from SR. Regardless of how it arises, the DR is an assembly-incompetent species. Its formation is an example of an off-pathway process (dashed lines) that competes with on-pathway reactions (solid lines) leading to functional 20S.

Until now, two separate narratives described the assembly of the 20S proteasome. In one, bacterial α subunits do not form rings but likely form heterodimers with β subunits that assemble into half-proteasomes which then dimerize to form the 20S proteasome^31^,^32^,^33^. In the other, archaeal and eukaryotic α subunits form α-rings first; these template β subunit incorporation until a half-proteasome is formed, which then dimerizes^19^,^20^. Here we suggest that this dichotomy might not be necessary. Archaeal proteasomes can assemble along a pathway independent of α-ring formation, reminiscent of bacterial 20S assembly (Fig. 7).

The α-ring first view of proteasome assembly arose from observations demonstrating that archaeal and eukaryotic α subunits form rings on their own^19^,^34^,^35^,^40^,^41^. Stability of α-rings is partly due to extensive inter-subunit interactions mediated by α subunit H0 helices. Lacking the N-terminal extensions that contain H0 helices, β subunits cannot form rings by themselves and depend on α-rings to guide their assembly^10^,^19^,^20^. Many of these studies relied on bacterial expression of proteasome subunits. This continues to be a valuable approach because one can generate subunits in isolation, in combination with other subunits, and as both wild-type and mutant versions, without the need to worry about interference from endogenous 20S, which *E. coli* lacks. Using recombinant α subunits from the archaeon *M. maripaludis*, which form single (SR) and double (DR) α-rings (Fig. 1), we show that highly conserved charged residues at the α–α subunit interface are important for α-ring stability, likely through the formation of stabilizing salt-bridges. The K59E and R88D α subunit mutants do not form any detectable SR (Fig. 2) yet both efficiently assemble into functional 20S proteasomes (Figs 3 and 4) via a pathway that involves direct formation of half-proteasomes (Figs 5 and 6), probably from αβ heterodimers (Supplementary Fig. S9).

Assembly of recombinant α subunits into DR had been documented^19^,^34^,^35^. The implicit assumption was that DR arose from SR, yet this was never explored. Here we show that the α (K59E) mutant, which does not form any detectable SR, is able to generate some DR (Fig. 2). This suggests that DR can form independently of SR. The significance of this observation is made clear by our cross-linking data showing that DR are structurally analogous to half-proteasomes; both types of double rings interact via H1 α helices (Fig. 1, Supplementary Fig. S1, and^10^). This quaternary structure for DR was foreshadowed by cryoEM analysis^10^,^19^ but our study presents the first biochemical confirmation of this arrangement. To form DR without first forming SR, α subunits need to pair *in trans* (i.e. using the H1-helix-based surfaces used to hold two α rings together). Since α and β subunits share the same structure, and interact via H1 helices, this *trans* pairing would be analogous to the formation of αβ heterodimers that give rise to half-proteasomes in bacteria^33^ and probably now in archaea. The structural similarities between DR and half-proteasomes, which this study confirms, suggest that direct α-subunit-to-DR assembly mimics the direct α-subunit-to-half-proteasome assembly, with both occurring independently of SR. Yet these similarities remained elusive, until now.

As the direct precursor to 20S CP, the half-proteasome is an important intermediate in CP assembly. Having more than one pathway to reach the half-proteasome could be advantageous in case one is compromised. The idea of alternative pathways for proteasome assembly is supported by studies which showed: that paralogous β subunits in mammalian immune cells are incorporated in a different order than their constitutive counterparts^42^,^43^,^44^; that deletion of an assembly factor in yeast results in simultaneous production of both normal CP and alternate versions in which a second copy of α4 replaces the endogenous copy of α3^23^; and that the 19S regulatory particle can assemble via pathways dependent on^45^,^46^,^47^,^48^, and independent of 49, a pre-existing 20S proteasome. Consequently, the concept of a single linear assembly “pathway” for the proteasome should perhaps be updated to an assembly “network” consisting of several pathways leading to the formation of this complex. There are ~33 different proteins that make up the eukaryotic proteasome. In addition to productive pathways leading to its formation, there will also be unproductive pathways giving rise to assembly-incompetent (i.e. dead-end) complexes. The DR may be one such complex. DR and various DR-like species have been postulated to be dead-end complexes in eukaryotes *in vivo*^22^,^26^,^50^. Archaeal proteasomes are compositionally simpler, but we show here that archaeal DR are poor substrates for CP formation (Fig. 6) and thus likely candidates for dead-end complexes.

If multiple assembly pathways are possible, determining the extent to which each pathway is populated *in vivo*, and how unproductive pathways leading to dead-end complexes are avoided, remains to be determined. Kinetics and thermodynamics governing subunit association are important; pairings that occur quickly and/or produce stable intermediate complexes will be favored^36^. According to the updated assembly model (Fig. 7), α subunits can assemble with each other *in cis*, leading to the formation of SR, or with β subunits, leading to the half-proteasome, or with each other *in trans*, leading to the unproductive DR. We observe DR formation under lysate mixing but not coexpression. This argues that the pathway leading α subunits to DR can be suppressed if conditions ensure that the SR and/or half-proteasome pathways occur faster. This is not the case during lysate mixing which artificially creates a low subunit concentration condition that slows assembly and thus allows the DR pathway to become populated. Besides kinetics and thermodynamics, dedicated assembly factors^22^,^26^,^50^ and post translational modifications^51^ will be shown to play increasingly important roles in shepherding assembling subunits onto productive pathways, and away from non-productive ones.

Our data do not question the importance of the SR to archaeal 20S assembly, as demonstrated by others^19^,^41^. Nor do our data establish the extent to which SR-dependent and SR-independent assembly occurs *in vivo*. However, our findings that archaeal 20S proteasomes can assemble along an SR-independent pathway, reminiscent of bacterial 20S assembly, suggest a path toward a clearer understanding of proteasome evolution. Unlike eukaryotes and archaea, the proteasome has a limited distribution in bacteria; it has been argued that a horizontal gene transfer (HGT) from archaea endowed these limited lineages with proteasomes^15^. Under the current dichotomy of assembly, one is forced to argue that bacterial 20S proteasomes must have lost their ability to assemble like archaeal proteasomes (SR-dependent pathway) and gained an entirely new assembly mechanism (SR-independent pathway) soon after HGT from the archaeal donor. Our results suggest that this ancestral archaeal donor assembled its 20S along both SR-dependent and SR-independent pathways, as its descendant *M. maripaludis* can today (at least *in vitro*). Therefore, the bacteria that received the proteasome from this donor would only need to lose the SR-dependent pathway while retaining the SR-independent pathway; no gain of function change is required. This is a more parsimonious explanation for the evolution of bacterial proteasome assembly, and is supported by structural data showing less surface area buried between α subunits to help stabilize a bacterial α ring^9^,^32^. The conserved CP architecture across all domains of life belies common assembly mechanisms that we suggest are conserved across evolutionary time. It will now be interesting to determine if eukaryotic 20S proteasomes also retain an SR-independent assembly pathway.

## Methods

Experiments in all figures were performed a minimum of three times using independently prepared protein samples.

### Creation of plasmids and mutant constructs

Plasmids used in this study are listed in Supplementary Table S1. DNA fragments encoding archaeal α and β subunits were cloned by PCR from *Methanococcus maripaludis* S2 genomic DNA kindly provided by John Leigh (University of Washington). Where indicated, primers were designed to incorporate C-terminal hexahistidine tags (his-tag). DNAs were subcloned into pET42 vector for expression in bacteria. Construction of polycistronic expression plasmids enabling the coexpression of archaeal α and β subunits was carried out as described^28^. Mutagenesis was carried out by PCR using the Quickchange method and kit (Stratagene). Automated DNA sequencing was used to verify all constructs.

#### Protein expression and isolation from bacteria

Plasmid transformation into *Escherichia coli* BL21 cells, subsequent induction of protein synthesis by IPTG, and harvesting of the cultures were performed as described^23^,^28^. Frozen cell pellets were thawed on ice and resuspended in 0.6 ml of Buffer A (50 mM HEPES-NaOH, pH 7.5, 0.3 M NaCl, and 5 mM MgCl_2_) supplemented with 2 mM Pefabloc, 0.3 mg ml^–1^ lysozyme, 10 μg ml^–1^ DNase I and 0.1% (v/v) Triton X-100. The suspensions were lysed by shaking at 30 °C for 30 min. The resulting total crude lysate was centrifuged at 10,000 × *g* for 10 min at room temperature to separate soluble and insoluble material. The soluble material was applied to 50 μl of equilibrated immobilized cobalt affinity resin (ICAR) (Talon resin; Clontech), incubated for 1 hour and centrifuged at 700 × *g* for 5 min. The resin beads were washed 2 times with 1 ml of Buffer A, 2 times with 1 ml of Buffer B (Buffer A supplemented with 5 mM imidazole), and 1 time with 1 ml of Buffer C (Buffer A supplemented with 10 mM imidazole). Each wash step was carried out with gentle rocking for 5 min at 4 °C, followed by centrifugation at 700 × *g* for 5 min to pellet the resin. His-tagged proteins were eluted in 600 μl of Buffer E (Buffer A supplemented with 200 mM imidazole). Following purification, protein samples were desalted by serial centrifugation as described^28^. Prior to gel electrophoresis or size exclusion chromatography, protein concentrations were measured using the BCA Assay (ThermoScientific). For lysate mixing experiments, total crude lysates of desired samples were mixed and incubated at 37 °C with slow shaking for 30 min. Following incubation, mixed total crude lysates were separated into soluble and insoluble fractions as described above and subjected to protein purification by ICAR.

### Polyacrylamide gel electrophoresis

Equal amounts of protein (10 μg or 20 μg) were mixed with 5× nondenaturing sample buffer (0.5 M Tris-HCl, pH 8.8, 50% (v/v) glycerol, traces of bromophenol blue). Samples were subjected to analysis by nondenaturing gel electrophoresis as described^23^,^28^ except 4–15% gradient, and 5–10% gradient gels were used as indicated in the figure legends. All gels were lab poured except for the 4–15% gradient gels which were precast Mini-PROTEAN TGX gels (BioRad). Aliquots of native high molecular weight marker mix for nondenaturing gel electrophoresis (GE Healthcare) were mixed with 5× nondenaturing sample buffer and loaded along with the protein samples. The electrophoretic run was carried out at 55 V and 4 °C until the dye front ran off the gel. Where indicated, following electrophoresis, nondenaturing gels were subjected to substrate overlay assay using the fluorogenic substrate Suc-LLVY-AMC (Enzo) to visualize the peptidase activity of the proteasome on a UV transilluminator^28^ and then stained with GelCode blue (ThermoScientific). Aliquots of samples analyzed by nondenaturing gel electrophoresis were mixed with 5× SDS sample buffer and separated on 12% SDS-PAGE as indicated in the figure legends.

### Cross-linking analysis

For experiments utilizing engineered cysteine mutant α subunits, no cross-linking and/or oxidizing agents were added to the samples to induce disulfide formation. Experimental conditions during expression, lysis, and ICAR purification were sufficiently oxidizing to allow disulfide bonds to form. Purified proteins (20 μg) were analyzed by nondenaturing PAGE as described above. Bands of interest were excised from the gel, cut into small pieces, and incubated overnight at 4 °C in 1× SDS sample buffer without DTT in order to allow proteins to elute. The supernatants containing the eluted proteins were analyzed by 12% SDS-PAGE under non-reducing conditions and stained with the Pierce Silver Stain Kit (ThermoScientific). Where indicated, DTT was added back to some aliquots prior to electrophoresis.

### Size exclusion chromatography

Wild-type and mutant α subunits (780 μg) were loaded on to a HiPrep Sephacryl S-300 HR column (GE Healthcare) coupled to an AKTA Prime Plus chromatography system (GE Healthcare). Elution profiles were analyzed using Prime View evaluation software. The column was equilibrated with Buffer D (25 mM Tris-HCl, pH 7, 150 mM NaCl), the flow rate was 0.8 ml min^−1^, and 3 ml fractions were collected. Calibration of the column was carried out using 360 μg of each of six molecular weight standards (Serva). Aliquots (15 μl) of sizing column fractions were mixed with 5× SDS sample buffer and analyzed by 12% SDS-PAGE followed by staining with GelCode blue. In addition, aliquots (50 μl) of sizing column fractions were mixed with 5× nondenaturing sample buffer and analyzed by nondenaturing 4–15% gradient precast gels followed by staining with Imperial Stain (ThermoScientific) or Pierce Silver Stain Kit (ThermoScientific). In experiments requiring the pooling of sizing column fractions, the indicated fractions were combined and concentrated down to a volume of 0.6 ml using Pierce Protein Concentrators, 9K (ThermoScientific). These pooled and concentrated fractions were then mixed with crude lysates of BL21 cells expressing untagged archaeal β subunits. Proteins were repurified by ICAR and analyzed by native PAGE and substrate-overlay assay as described above.

## Additional Information

**How to cite this article**: Panfair, D. *et al.* Alpha-ring Independent Assembly of the 20S Proteasome. *Sci. Rep.* **5**, 13130; doi: 10.1038/srep13130 (2015).

## Supplementary Material

Supplementary Information

Supplementary Full Gels
