# Supplementary material for: Alpha-ring Independent Assembly of the 20S Proteasome
Source: Sci Rep. 2015 Aug 19;5:13130. doi: 10.1038/srep13130 (PMC4541365; doi:10.1038/srep13130)
Supplement: Supplementary Information [file srep13130-s1.pdf]

Supplementary Information for:

Alpha-ring Independent Assembly of the 20S Proteasome

Dilrajkaur Panfair, Aishwarya Ramamurthy, and Andrew R. Kusmierczyk

Table S1

Figures S1 to S9

Supplementary Note

Supplementary References

Supplementary Table S1: Plasmids used in this study

Name	Genotype	Source
AKB191	pET42 psmA-his	supp. ref. 1
AKB254	pET42 psmA (R88D)-his	This study
AKB257	pET42 psmA (K59E)-his	This study
AKB946	pET42 psmB	This study
AKB950	pET42 psmB Δpro	This study
AKB951	pET42 psmB (T1A)	This study
AKB952	pET42 psmB (R166W)	This study
AKB464	pET42 psmA-his psmB	supp. ref. 1
AKB600	pET42 psmA (Q99C)-his	This study
AKB628	pET42 psmA-his psmB Δpro	supp. ref. 1
AKB572	pET42 psmA-his psmB (T1A)	supp. ref. 1
AKB573	pET42 psmA-his psmB (R166W)	This study
AKB706	pET42 psmA (A98C)-his	This study
AKB707	pET42 psmA (M100C)-his	This study
AKB708	pET42 psmA (Δcys)-his	This study
AKB709	pET42 psmA (Q99C Δcys)-his	This study
AKB727	pET42 psmA (K59E)-his psmB	This study
AKB943	pET42 psmA (K59E)-his psmB (R166W)	This study
AKB944	pET42 psmA (K59E)-his psmB (T1A)	This study
AKB945	pET42 psmA (K59E)-his psmBΔpro	This study
AKB949	pET42 psmA (R88D)-his psmB	This study
AKB953	pET42 psmB-his	This study
AKB964	pET42 psmA (R88D)-his psmBΔpro	This study
AKB965	pET42 psmA (R88D)-his psmB (T1A)	This study
AKB966	pET42 psmA (R88D)-his psmB (R166W)	This study
AKB976	pET42 psmB (K29E)	This study
AKB988	pET42 psmB (K29E)-his	This study

psmA = α subunit from *Methanococcus maripaludis*
psmB = β subunit from *Methanococcus maripaludis*

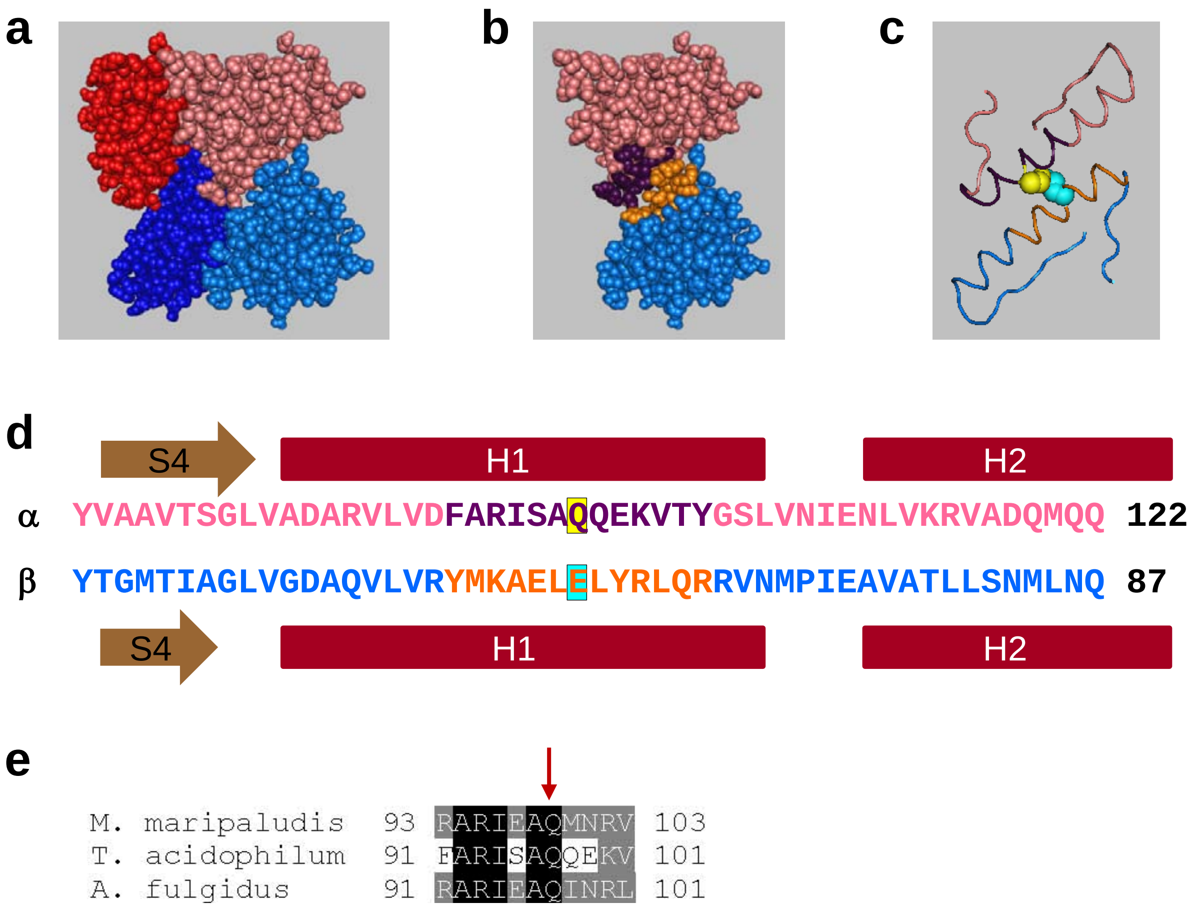

Supplementary Figure S1.

The H1 helices of α and β subunits contribute to inter-ring contacts.

Structural models based on the *T. acidophilum* crystal structure (1PMA) and generated with the program Cn3D. **(a)** Subunit contacts between two neighboring α (red/pink) and two β (blue) subunits viewed from inside the 20S cavity. **(b)** Subunit contacts and color scheme as in (a), with an α-β subunit pair isolated for emphasis. The C-terminal halves of helices H1 in each subunit are indicated in purple and orange. Close-up of H1 helices **(c)** and sequence alignment of relevant region **(d)** with color scheme as in (a,b). Glutamine 97 in the H1 helix of the *T. acidophilum* α subunit (yellow) is juxtaposed with the corresponding aligned residue (glutamate 62; teal) in the β subunit. Hence, if a second α subunit were to take the place of the β subunit, as hypothesized for a double α-ring, the indicated glutamine might be suitably positioned for an engineered disulfide cross-link. The equivalent position in the *M. maripaludis* α subunit is Q99 **(e)**.

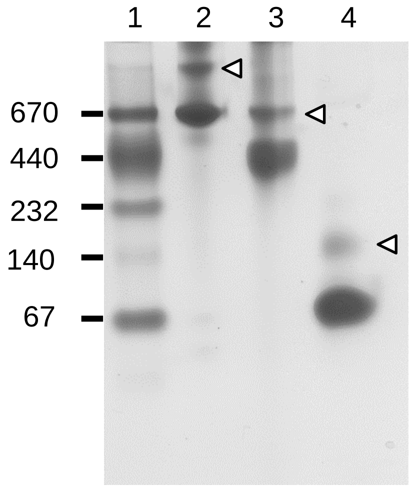

Lane 1: molecular size standards (kDa)
Lane 2: 670 kDa standard (Thyroglobulin)
Lane 3: 440 kDa standard (Ferritin)
Lane 4: 67 kDa standard (BSA)

Supplementary Figure S2.

Gel-induced higher order species artifacts.

In order to better visualize some lower-abundance species, native gels in this study were frequently heavily loaded with protein (10–20 μg per lane). When this was done, however, one could sometimes observe higher order species migrating above the band of interest. These are identified by white arrowheads in the main figures and they are gel-induced artifacts. Formation of gel-induced artifacts can be illustrated in the nondenaturing 4–15% gradient gel above, stained with GelCode blue. Lane 1 contains the molecular size standards used throughout this study. In lanes 2–4, some of these standards are heavily loaded and run individually. In addition to the correctly running major band, the heavily loaded samples exhibit higher order species to varying degrees (white arrowheads).

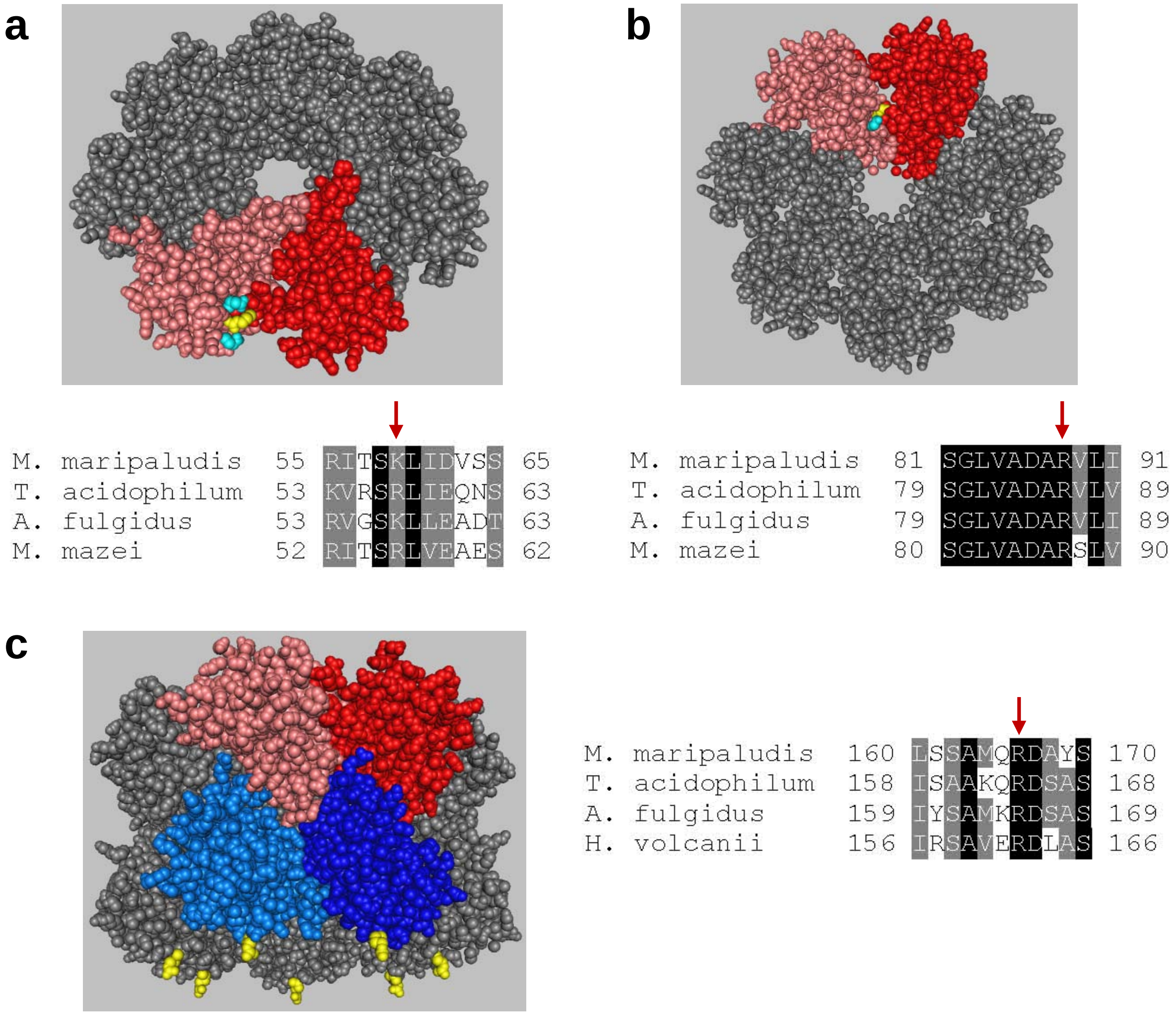

Supplementary Figure S3.

Residues selected for mutagenesis.

Space filling models based on the *T. acidophilum* crystal structure (1PMA) and generated with the program Cn3D. **(a,b)** Highly conserved charged residues (yellow) in one α subunit (red) are juxtaposed with highly conserved residues of opposite charge (teal) located within 5 angstroms on the neighboring α subunit (pink). **(a)** Top view of α-ring with R57 of *T. acidophilum* (yellow). The equivalent position in *M. maripaludis* α subunit is K59. **(b)** Bottom view of α-ring with R86 of *T. acidophilum* (yellow). The equivalent position in *M. maripaludis* α subunit is R88. **(c)** Side view of the half-proteasome, angled upward, showing highly conserved β subunit arginine 164 (yellow). Two α and β subunits are colored for reference. Numbering in the sequence alignment is based on the N-terminal catalytic threonine being assigned position1; hence in *M. maripaludis* the β subunit arginine is at position166.

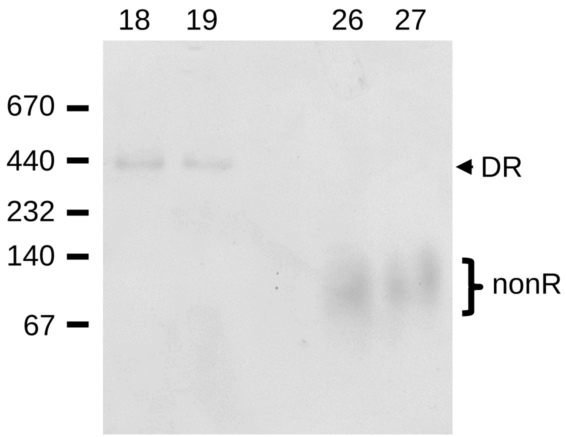

Supplementary Figure S4.

The K59E mutant can form some DR without apparently forming SR.

Following the fractionation of the α (K59E) mutant by size exclusion chromatography (**Fig. 2b** main text), aliquots of fractions 18, 19, 26, and 27 were analyzed by native PAGE on a nondenaturing 4–15% gradient gel shown here stained with Imperial Stain This analysis confirmed the assignment of the major peak in fractions 25–28 as the nonR peak, and the minor peak in fractions 17–19 as the DR peak (see main text). The data are consistent with the α (K59E) mutation interfering with the assembly of SR, yet still allowing some DR to form.

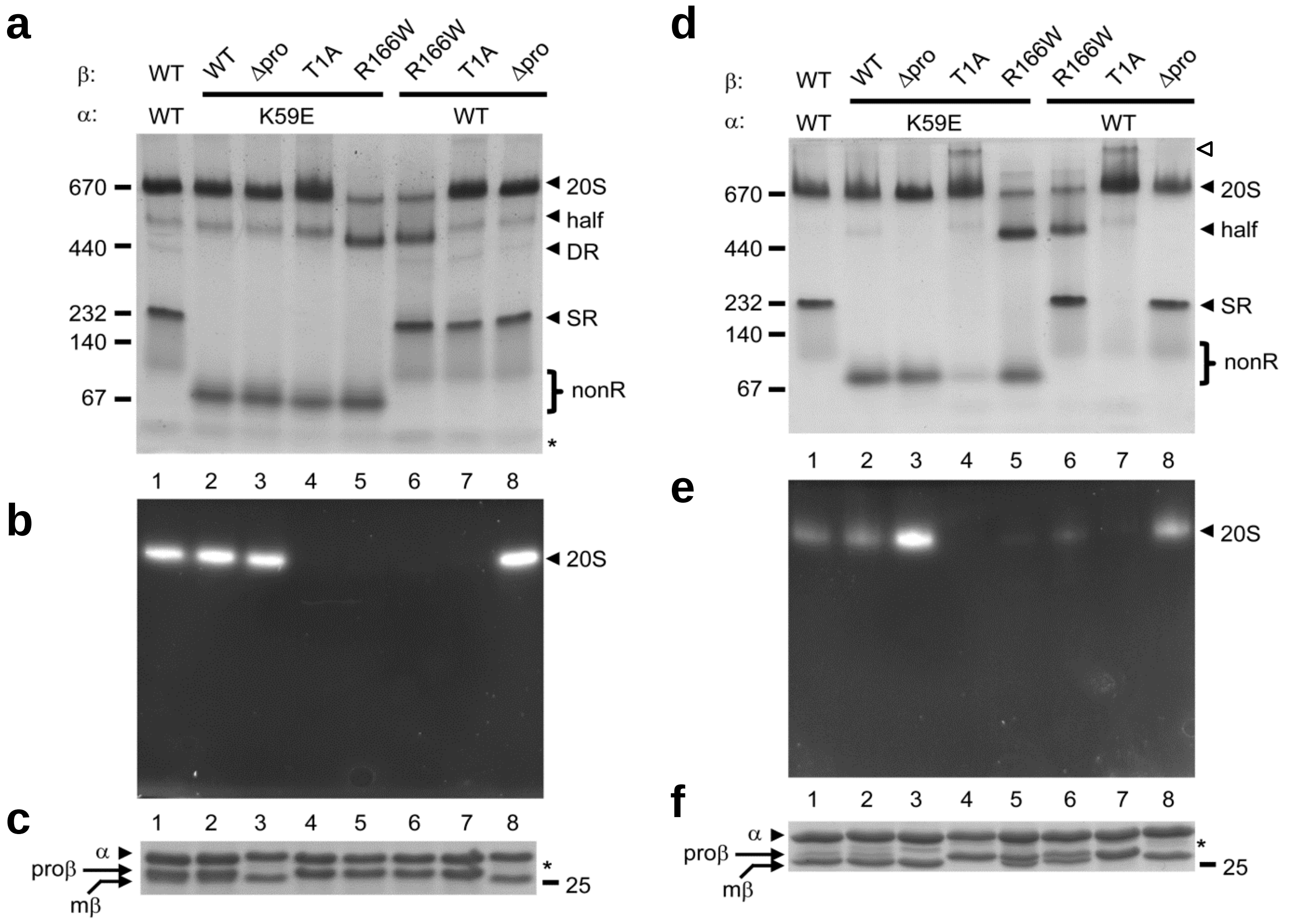

Supplementary Figure S5.

Assembly of archaeal proteasomes exhibits bacterial-like features.

The experiments described in the panels above use the α (K59E) mutant but are otherwise identical to those carried out with the α (R88D) mutant described in the main text (**Fig. 5**). Indeed, the figure legend to **Figure 5** applies here exactly, with K59E substituted for R88D. The results of this figure support the same conclusions drawn from the main text (**Fig. 5**) namely: (i) that archaeal 20S assembly can proceed along a bacterial-like pathway, independent of SR; (ii) that this SR-independent pathway still proceeds through a half-proteasome; (iii) that the SR-independent pathway does not depend on the β subunit propeptide; and (iv) that the same conclusions could be drawn regardless of the approach, lysate mixing versus coexpression, though the latter approach results in more efficient assembly (see main text pertaining to **Fig. 5**).

As stated in the main text, there are two minor differences between lysate mixing and coexpression approaches. These differences do not affect the aforementioned conclusions but they are worth noting here. First, in lysate mixing experiments, levels of the half-

Supplementary Figure S5 (continued)

proteasome were similar within wild-type α subunit samples (**Fig. 5a** and **Supplementary Fig. S5a**, lanes 1, 7, 8), and within mutant samples (**Fig. 5a** and **Supplementary Fig. S5a**, lanes 2, 3, 4), regardless of the β subunit propeptide variant employed. By contrast, this uniformity was absent during coexpression (see corresponding lanes in **Fig. 5d** and **Supplementary Fig. S5d**). This was likely due to slower assembly in lysate mixing where the rate limiting step might be (i) the association of α subunits into SR and/or (ii) the association of α and β subunits into half-proteasomes. Second, in coexpression experiments, the samples employing β (T1A) exhibited sharp decreases in SR and nonR (**Fig. 5d** and **Supplementary Fig. S5d**, lanes 4 and 7). By contrast, SR and nonR levels were constant in lysate mixing regardless of which β subunit variant was being used. This suggests that under coexpression conditions, where assembly is already more efficient than in lysate mixing, the T1A mutant is even more efficient at interacting with free subunits, and/or SR, than a wild-type β subunit. However, the reason for this is unknown to us.

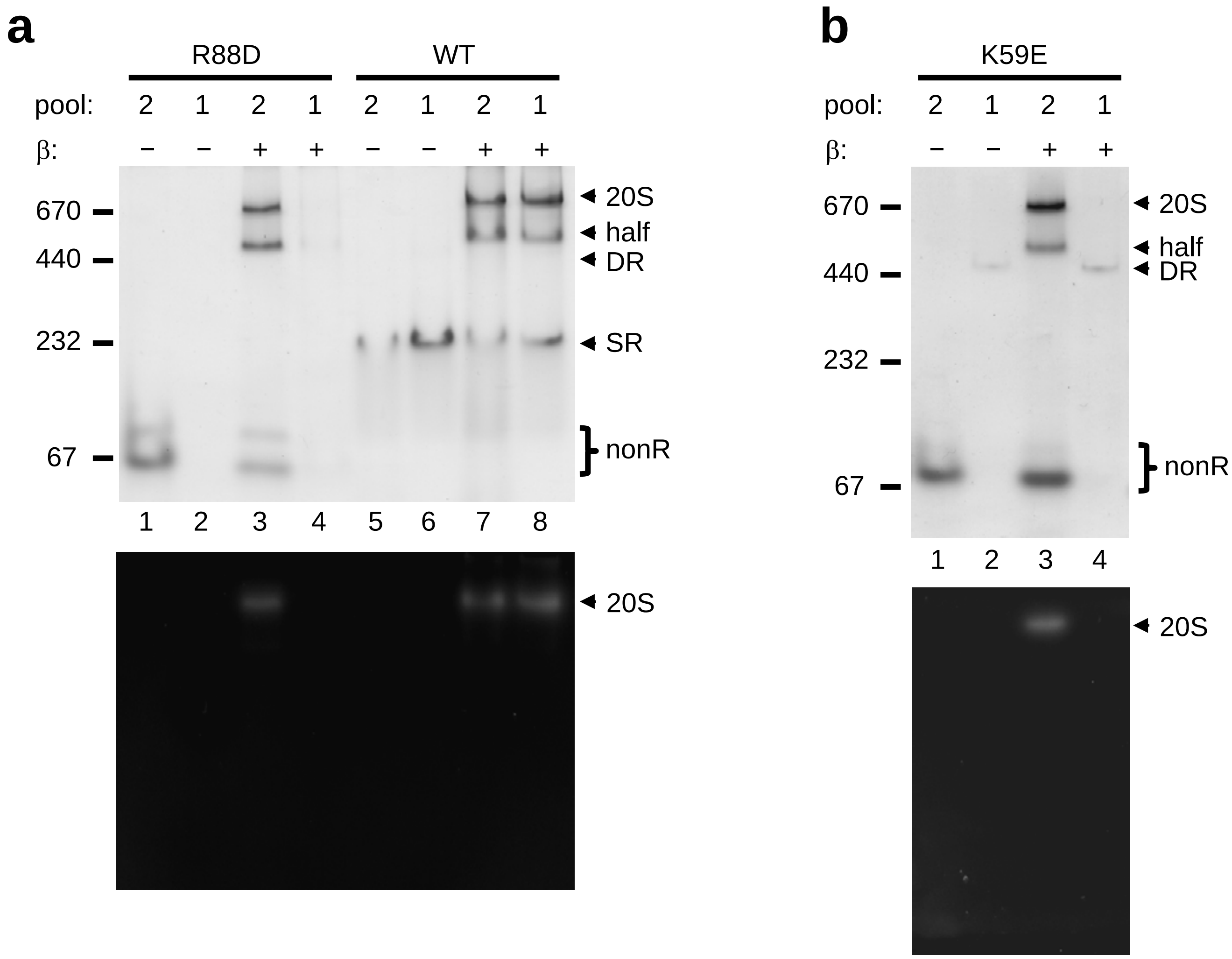

Supplementary Figure S6.

Ring independent assembly of archaeal 20S proteasomes.

This is another iteration of the same experiment as described in **Figure 6**. The difference is that in this version, the isolated pool 1 and pool 2 samples for each of the α-his proteins (**a**, wild-type and R88D; **b**, K59E) were split in half. One half was mixed with an equal volume of lysate from *E. coli* expressing wild-type archaeal β subunits (+) and the other half was not (−). Following incubation to allow assembly to occur, the proteins were repurified by ICAR and equal volumes of each eluate were electrophoresed on a nondenaturing 5–10% gradient gel. Immediately prior to GelCode staining (top panels), the polyacrylamide gel was overlaid with buffer solution containing the fluorogenic peptide substrate Suc-LLVY-AMC to detect peptidase activity (bottom panel). Black arrowheads denote the positions of assembled 20S core particle (20S), half-proteasome (half), double α-ring (DR) and single α-ring (SR). The position of α subunit species that do not assemble into any ring (nonR), and are mostly free α subunits, is shown with a bracket. The migration of several molecular size standards (in kDa) is indicated.

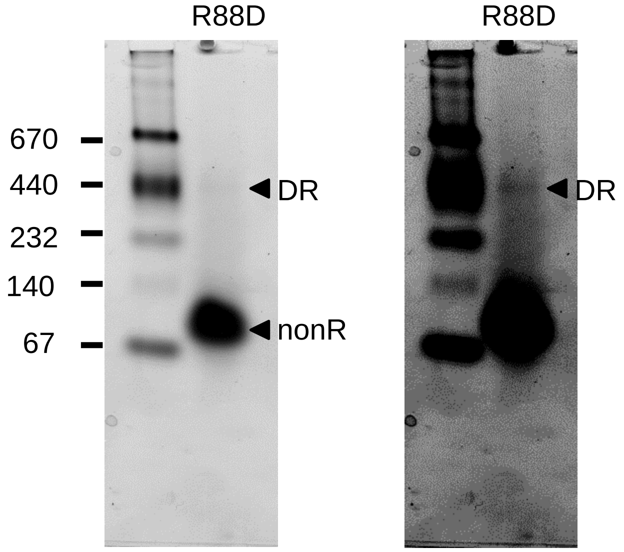

Supplementary Figure S7.

On the severity of the R88D mutation.

The α (K59E) mutant does not appear capable for forming any detectable SR, though it does form some DR, while the α (R88D) mutant appears to form neither SR nor DR (**Figs. 2,3**). Both mutants exist primarily as nonR (mostly free α subunits) species. Although the effect of the R88D mutation on ring assembly is profound, it is not absolute. When purified α (R88D) protein is heavily overloaded on a nondenaturing 4–15% gradient gel, one can begin to discern a very faint DR band (left panel). Adjusting the brightness and contrast of this image makes this band a bit easier to visualize (right panel). No SR band is ever seen. Hence, the α (R88D) mutation can be thought of as a more extreme version of the α (K59E) mutation i.e. neither forms SR but α (R88D) is much more severe in its effect on DR.

Since the levels of DR in the α (R88D) mutant sample are almost but not quite zero, this can help explain why pool 1 from this mutant also gave rise to barely-perceptible levels of 20S (**Fig. 6a**, lane 4 and **Supplementary Fig. S6a**, lane 4). Although present at much lower levels than the α (K59E) DR, the α (R88D) DR is inherently less stable and will dissociate more readily into assembly competent nonR (mostly free α subunits). Because such a large amount of protein is loaded onto the Sephacryl S-300 column (780 μg), there should be enough DR present in pool 1 that, upon concentration and mixing with β-subunit-containing lysates, vanishingly small but detectable levels of 20S species will form. Regardless, these tiny levels of 20S in the pool 1 samples do not alter the main point (**Fig. 6** and **Supplementary Fig. S6**) that free α subunits (pool 2) can serve as the starting point for efficient CP assembly along a pathway that does not require SR to form.

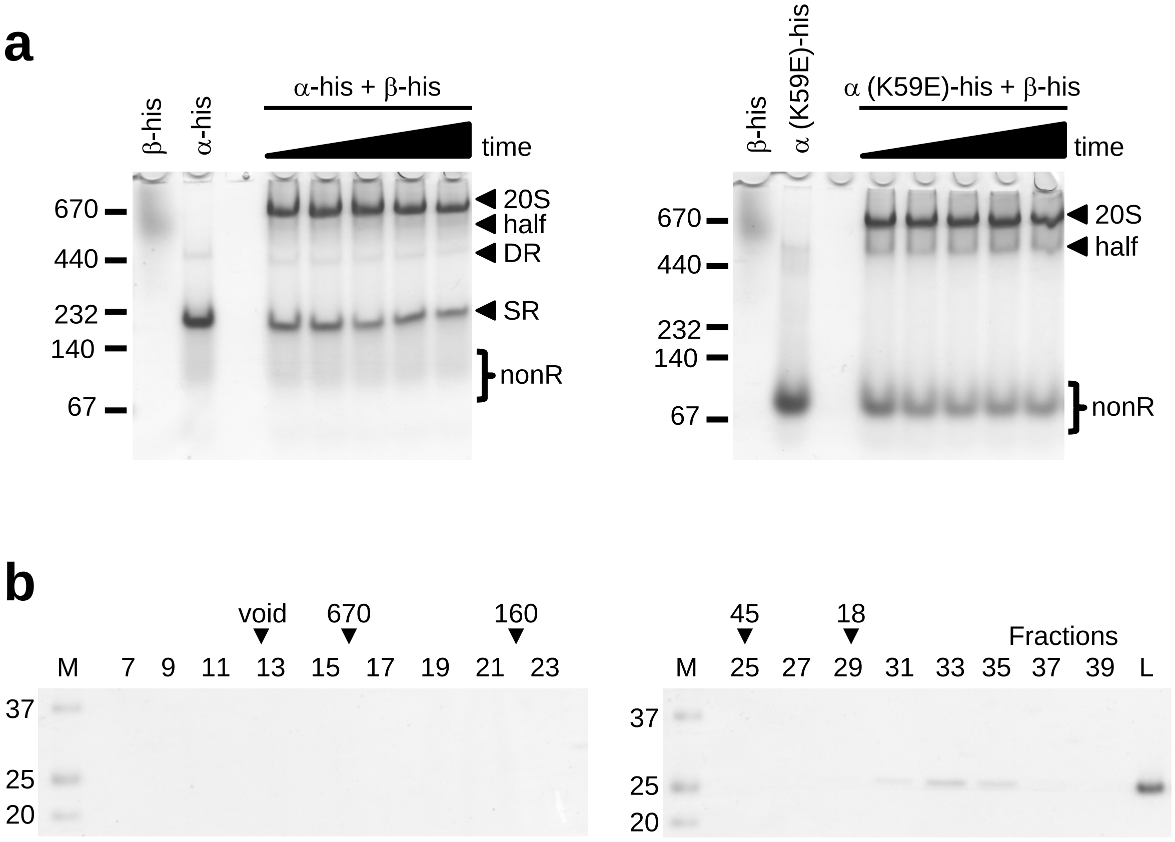

Supplementary Figure S8.

Rapid assembly of 20S proteasomes following subunit mixing.

To visualize additional assembly intermediates, we sought to carry out mixing experiments using separately expressed wild-type, or mutant, α-his and wild-type β-his subunits that were first purified via ICAR. (**a**) Purified wild-type, or K59E mutant, α-his (10 μg) was mixed with purified wild-type β-his (10 μg) and incubated for increasing amounts of time (0 to 20 min) prior to loading onto a nondenaturing 5–10% gradient gel. The dead time of the experiment was ~8 min. This is the time it takes to withdraw an aliquot of the assembly mixture, add nondenaturing sample buffer, load the sample, turn on the voltage, and have the sample enter the gel. Assembly can continue during the dead time because the sample is always under nondenaturing conditions and it is only when protein enters the gel (and free α and β subunits begin to separate from each other) that assembly is no longer happening. The data suggest that most of the assembly observed occurred during the dead time. Black arrowheads denote the positions of assembled 20S core particle (20S), half-proteasome (half), double α-ring (DR) and single α-ring (SR). The position of α subunit species that do not assemble into any ring (nonR), and are mostly free α subunits, is shown with a bracket. The migration of several molecular size standards (in kDa) is indicated. As stated in the main text, no new assembly intermediates (i.e. αβ heterodimers) were evident in mixing experiments. This could be because αβ heterodimers are transient and very rapidly assemble into half-proteasomes, or because αβ heterodimers are not stable enough to survive electrophoresis, or both.

Supplementary Figure S8 (continued).

While purified α-his subunits (wild-type or mutant) resulted in distinct species when separated by nondenaturing PAGE, purified β-his subunits produced a slowly migrating smear (**a**)**.** We attribute this to the high predicted isoelectric point for β-his (pI = 8.39). Since the pH of the nondenaturing gel is 8.8, β-his may not be appreciably negatively charged at this pH to easily enter the gel. In support of this, we fractionated purified β-his by size exclusion chromatography. (**b**) ICAR-purified recombinant β-his subunits were subjected to size exclusion chromatography on a Sephacryl S-300 column. Aliquots of every other fraction were analyzed by 12% SDS-PAGE followed by staining with Imperial Stain. Black arrowheads indicate the column void volume and the elution peaks of molecular size standards (in kDa). M, molecular size standards (size in kDa indicated at left). L, aliquot of the sample load. Purified β-his elutes late as a single peak. No β-his was observed near the void volume or in high molecular weight fractions, consistent with purified β-his behaving as a monomer. The slightly later elution of the β-his protein (predicted M_r of 24.6 kDa) relative to the molecular size standards could reflect weak affinity of the β-his subunit for the Sephacryl resin. At the near neutral pH of the size exclusion column, the basic β-his protein (predicted pI = 8.39) may weakly bind to the low levels of carboxylates in the Sephacryl matrix thereby slowing its elution.

a

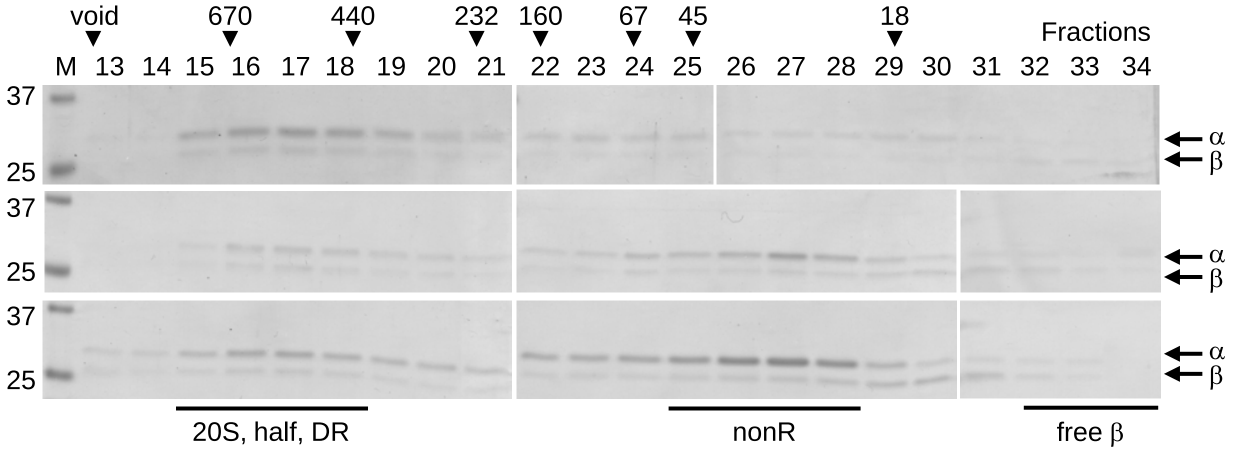

Lysates mixed in each panel in (a)			
**i.**	wild-type α-his	+	wild-type β
**ii.**	α (K59E)-his	+	wild-type β
**iii.**	α (K59E)-his	+	β (K29E)

b

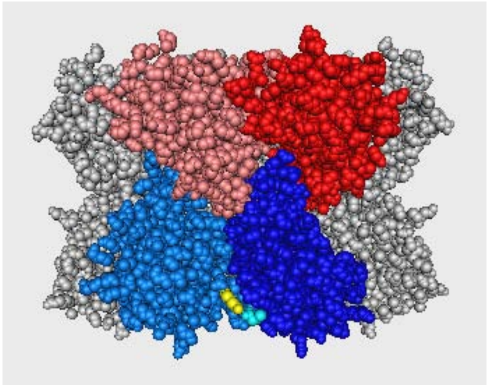

Supplementary Figure S9.

SR-independent assembly likely proceeds via αβ heterodimer formation.

(**a**) Proteasome assembly was initiated by mixing equal volumes of lysates from cells separately expressing the indicated α-his and β subunits. After 30 minutes, proteins were purified by ICAR and loaded onto a Sephacryl S-300 size exclusion column and 3 ml fractions were collected. Aliquots (50 μl) of the indicated fractions were analyzed by three 12% SDS-PAGE gels and stained with Imperial stain. Black lines delineate the position of the 20S peak (also containing half-proteasomes and DR) as well as the α subunit nonR peak and free β subunit peak. We note that both wild-type (**Supplementary Fig. S8**) and mutant β subunits (K29E; not shown) elute in fractions 32-34 when expressed on their own. The locations of the column void volume and the elution peaks of the indicated molecular size standards (in kDa) are indicated with black arrowheads. M, molecular size standards (size in kDa indicated at left). Panels (ii) and (iii) show progressively increasing amounts of β subunits eluting in fractions 25-30, consistent with αβ heterodimer formation. (**b**) Side view of the half-proteasome based on the *T. acidophilum* crystal structure (1PMA) and generated with the program Cn3D. Two α and β subunits are colored for reference. A conserved lysine (K29; yellow) in one β subunit is shown juxtaposed with a conserved residue of opposite charge (teal) located within 5 angstroms on the neighboring β subunit. Numbering is based on the N-terminal catalytic threonine being assigned position1; in *M. maripaludis* this β subunit lysine is also at position 29.

Supplementary Note

On the function of β subunit propeptides.

In **Figure 5**, we demonstrate that the archaeal β subunit propeptide is dispensable for CP assembly even when archaeal α subunits cannot form rings; assembly occurs with comparable efficiency with or without the propeptide. The bacterial β subunit propeptide is also not required for CP assembly[2,3] hence the dispensable nature of the propeptide is conserved in archaeal and bacterial CP assembly. However, this is not to argue that the purpose of the propeptide is identical in both. A number of differences are worth discussing.

The cis α–α subunit interface in bacterial proteasomes is much smaller than in eukaryotic/archaeal counterparts[4,5]. This likely contributes to the inability of bacterial α subunits to form stable α rings on their own[2], though dimers and trimers are apparently possible[6]. Consequently, bacterial β subunit propeptides are large in part to provide this missing contact surface and help stabilize bacterial α rings[5]. Consistent with this, in the absence of the β subunit propeptide, assembly of CP from *Rhodococcus erythropolis* is very inefficient[2]. Interestingly, assembly of CP from *Mycobacterium tuberculosis* is not affected by the absence of its corresponding propeptide[3]. This argues that the "stabilization-of-α-subunit-interface" function, while important, likely cannot be the only role for a bacterial β subunit propeptide in assembly. The visualization of the *Mycobacterium* propeptides outside (and below) the β-ring in a half proteasome[7], and their ability to negatively impact assembly[3], are consistent with this view.

Unlike bacterial propeptides, archaeal β subunit propeptides are much shorter. Clearly, the "stabilization-of-α-subunit-interface" function is not required by archaeal proteasomes since their α subunits can form stable α-rings independently. Also, archaeal β subunit propeptides are completely dispensable for assembly[8-10]. Our data suggests that assembly is equally efficient in their presence or absence, even when archaeal α subunits cannot form rings. So what is their role? One possible function is to ensure that the active site threonine is only exposed upon completion of assembly where it is safely enclosed within the central cavity. Numerous observations that propeptide processing is coupled to assembly, for proteasomes from all three domains of life, support this view[6,7,11-14]. Related to this function is the finding that the active site threonine can also be inactivated by N-acetylation if exposed to the cytosolic milieu[15]; a propeptide would again serve in a protective role until assembly is nearly complete. A third possible function is that propeptides can allosterically convey assembly status. We demonstrated the ability of PbaA, the putative archaeal ortholog of the Pba1-Pba2 assembly factor that binds to the outer α-ring surface, to preferentially bind to propeptide-containing intermediates[1]. This binding became progressively weaker with increased β subunit processing and let us to propose a "safety" function for this assembly factor: by recognizing propeptide-containing intermediates, assembly factors like PbaA and Pba1-Pba2 could prevent the premature association of activators (such as PAN in archaea, or RP in eukaryotes) to incompletely assembled CP[1]. This safety function was recently confirmed for Pba1-Pba2 in yeast[16].

All three of these functions are not mutually exclusive, but all of them can be satisfied by a small propeptide like those found in archaea. Since all three of these functions reduce the incidence of undesirable events, this could help explain the non-essential nature of these propeptides, namely: assembly per se is not affected in their absence, only the incidence of (undesirable) side-reactions would increase. Most pertinent to this study, all three of these functions are compatible with an α-ring dependent and an α-ring independent assembly

mechanism, both of which we show to be possible for the archaeal CP. Other functions beyond those suggested here may also exist, and may come to light in future studies.

On the formation of αβ heterodimers.

In **Supplementary Figure S9**, and the accompanying main text, we describe experiments aimed at determining if SR-independent assembly of archaeal proteasomes can proceed through the formation of αβ heterodimers. Lysate mixing was used to initiate proteasome assembly and, after 30 min, an assembly reaction was subjected to ICAR and the purified proteins loaded onto a size exclusion column. Fractions 15–18 contained coeluting α and β subunits and corresponded to assembled proteasomes (and other large species). Fractions 25–30 also contained coeluting α and β subunits and we present arguments in the main text supporting our claim that these fractions could contain the putative αβ heterodimers. Here, we put forth additional arguments based on our data to support this claim.

First, the overlap between the nonR peak of α-his subunits and the presence of β subunits in fractions 25–30 would be expected given that nonR species (i.e. mostly free α subunits) are the immediate precursor to αβ heterodimers. Second, the only way β subunits should end up in fractions 25–30 (or any fractions for that matter) is via complex formation with α-his subunits because free untagged β subunits do not bind to the metal-affinity resin (ICAR purification) that immediately precedes the size exclusion separation. Finally, the elution of β subunits in fractions 25–30 is not observed when β subunits are fractionated on a size exclusion column in the absence of α subunits; free β subunits elute in fractions 32–34 (**Supplementary Fig. S8**). This shift in elution profile (from fractions 32–34 to fractions 25–30) implies a higher molecular mass and thus complex formation with α subunits.

Taken together, our data support the existence of archaeal αβ heterodimers but they also do not rule out other interpretations (i.e. heterotrimers). However, it is important to note that this lack of absolute certainty is also the case with bacterial αβ heterodimers. A mass-spectrometry analysis has provided the first, and to date the only, physical evidence of bacterial αβ heterodimer formation[6]. However, all the αβ heterodimer species identified in that study contained truncated β subunits that lacked no fewer than 25 N-terminal amino acids of the 65 amino acid propeptide[6]. The authors themselves allow that an alternate explanation for these complexes is that they are “trapped” species, not capable of assembly[6].

The formation of αβ heterodimers is the most plausible explanation for how proteasome assembly can occur in the absence of SR formation. Data in bacteria[2,5,6] and now in archaea (this study) strongly support this view. However, one must also concede that a bona fide αβ heterodimer (consisting of a full length α subunit bound to a full length β subunit) functioning as an assembly-competent species remains to be unequivocally demonstrated for any proteasome.

Supplementary References

1. Kusmierczyk, A.R., Kunjappu, M.J., Kim, R.Y. & Hochstrasser, M. A conserved 20S proteasome assembly factor requires a C-terminal HbYX motif for proteasomal precursor binding. *Nat Struct Mol Biol* **18**, 622-9 (2011).
2. Zuhl, F., Seemuller, E., Golbik, R. & Baumeister, W. Dissecting the assembly pathway of the 20S proteasome. *FEBS Lett* **418**, 189-94 (1997).
3. Lin, G. et al. Mycobacterium tuberculosis prcBA genes encode a gated proteasome with broad oligopeptide specificity. *Mol Microbiol* **59**, 1405-16 (2006).
4. Hu, G. et al. Structure of the Mycobacterium tuberculosis proteasome and mechanism of inhibition by a peptidyl boronate. *Mol Microbiol* **59**, 1417-28 (2006).
5. Kwon, Y.D., Nagy, I., Adams, P.D., Baumeister, W. & Jap, B.K. Crystal structures of the Rhodococcus proteasome with and without its pro-peptides: implications for the role of the pro-peptide in proteasome assembly. *J Mol Biol* **335**, 233-45 (2004).
6. Sharon, M., Witt, S., Glasmacher, E., Baumeister, W. & Robinson, C.V. Mass spectrometry reveals the missing links in the assembly pathway of the bacterial 20 S proteasome. *J Biol Chem* **282**, 18448-57 (2007).
7. Li, D., Li, H., Wang, T., Pan, H. & Lin, G. Structural basis for the assembly and gate closure mechanisms of the Mycobacterium tuberculosis 20S proteasome. *EMBO J* **29**, 2037-47 (2010).
8. Maupin-Furlow, J.A., Aldrich, H.C. & Ferry, J.G. Biochemical characterization of the 20S proteasome from the methanoarchaeon Methanosarcina thermophila. *J Bacteriol* **180**, 1480-7 (1998).
9. Wilson, H.L., Ou, M.S., Aldrich, H.C. & Maupin-Furlow, J. Biochemical and physical properties of the Methanococcus jannaschii 20S proteasome and PAN, a homolog of the ATPase (Rpt) subunits of the eucaryal 26S proteasome. *J Bacteriol* **182**, 1680-92 (2000).
10. Zwickl, P., Kleinz, J. & Baumeister, W. Critical elements in proteasome assembly. *Nat Struct Biol* **1**, 765-70 (1994).
11. Chen, P. & Hochstrasser, M. Autocatalytic subunit processing couples active site formation in the 20S proteasome to completion of assembly. *Cell* **86**, 961-72 (1996).
12. Schmidtke, G. et al. Analysis of mammalian 20S proteasome biogenesis: the maturation of beta-subunits is an ordered two-step mechanism involving autocatalysis. *EMBO J* **15**, 6887-98 (1996).
13. Seemuller, E., Lupas, A. & Baumeister, W. Autocatalytic processing of the 20S proteasome. *Nature* **382**, 468-71 (1996).
14. Witt, S. et al. Proteasome assembly triggers a switch required for active-site maturation. *Structure* **14**, 1179-88 (2006).
15. Arendt, C.S. & Hochstrasser, M. Eukaryotic 20S proteasome catalytic subunit propeptides prevent active site inactivation by N-terminal acetylation and promote particle assembly. *EMBO J* **18**, 3575-85 (1999).
16. Wani, P.S., Rowland, M.A., Ondracek, A., Deeds, E.J. & Roelofs, J. Maturation of the proteasome core particle induces an affinity switch that controls regulatory particle association. *Nat Commun* **6**, 6384 (2015).
