# Supplementary material for: Alpha-ring Independent Assembly of the 20S Proteasome
Source: Sci Rep. 2015 Aug 19;5:13130. doi: 10.1038/srep13130 (PMC4541365; doi:10.1038/srep13130)

Supplementary Information for:

Alpha-ring Independent Assembly of the 20S Proteasome

Dilrajkaur Panfair, Aishwarya Ramamurthy, and Andrew R. Kusmierczyk

Uncropped Gels

Figure 1a
M = molecular size standards (kDa).

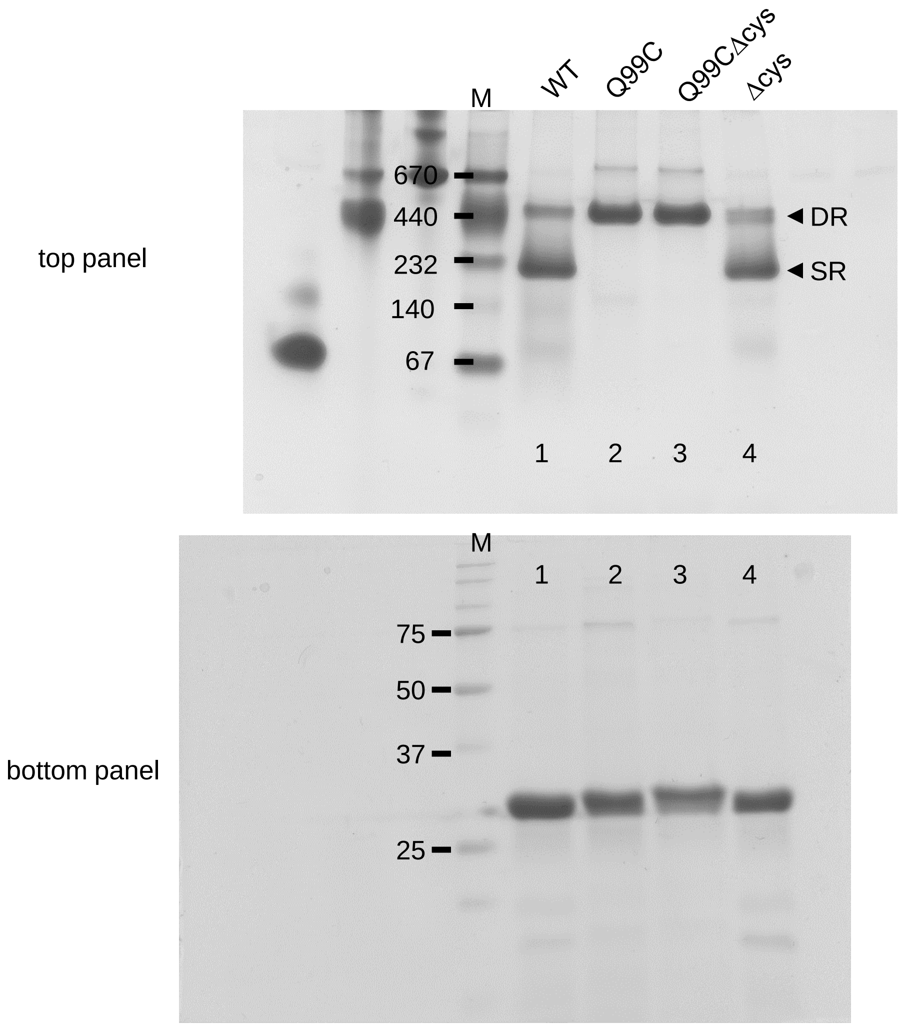

Figure 1b

These are the three panels of the composite sizing column figure for the wild-type.
M = molecular size standards (kDa).

left panel

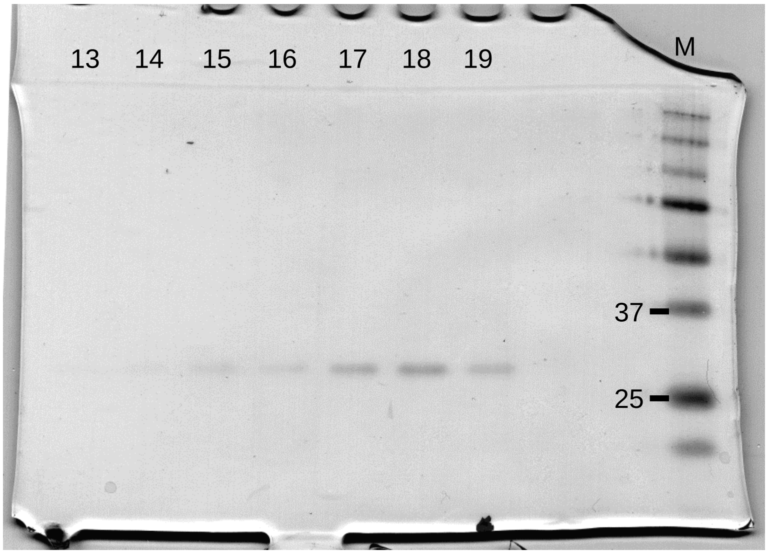

middle panel

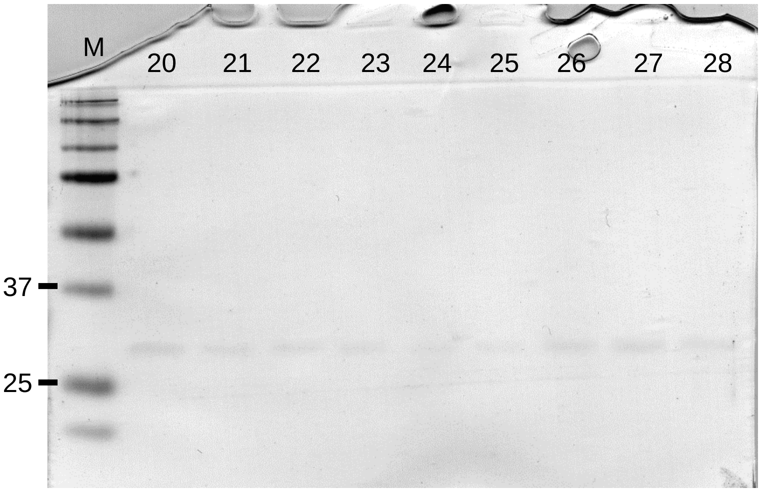

right panel

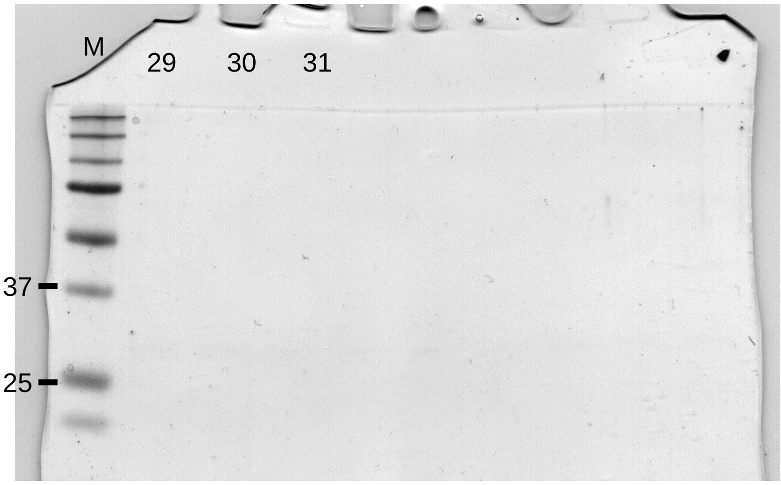

Figure 1b (continued)
This is the native gel from the "DR,SR" fractions of the K59E sizing column analysis. Dashed lines represent what is shown in actual figure. M = molecular size standards (kDa).

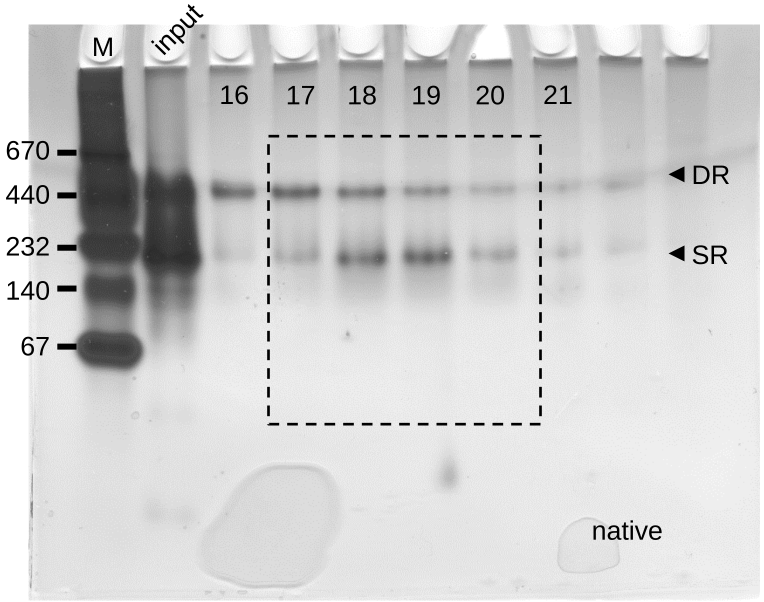

Figure 1c

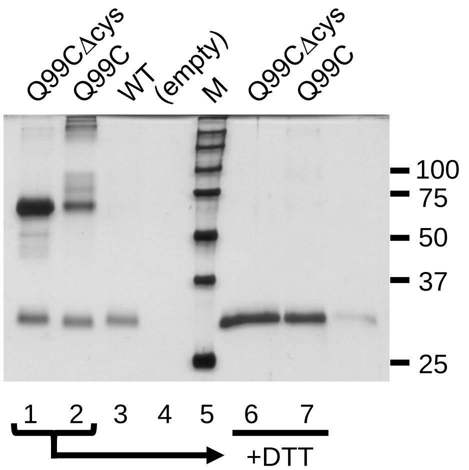

Figure 1d
M = molecular size standards (kDa)

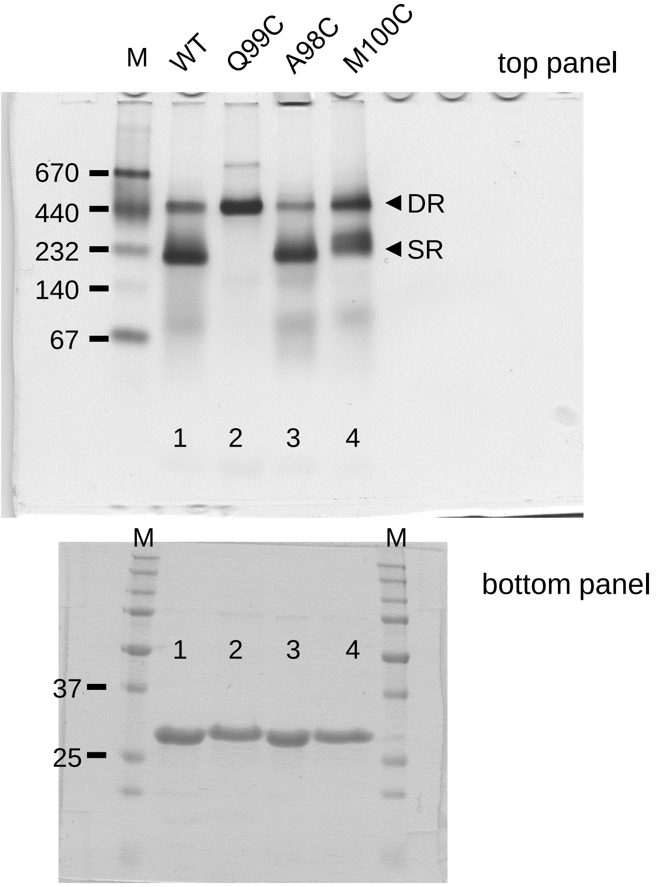

Figure 2a

Dashed line represent what is shown in the actual figure.

M = molecular size standards (kDa).

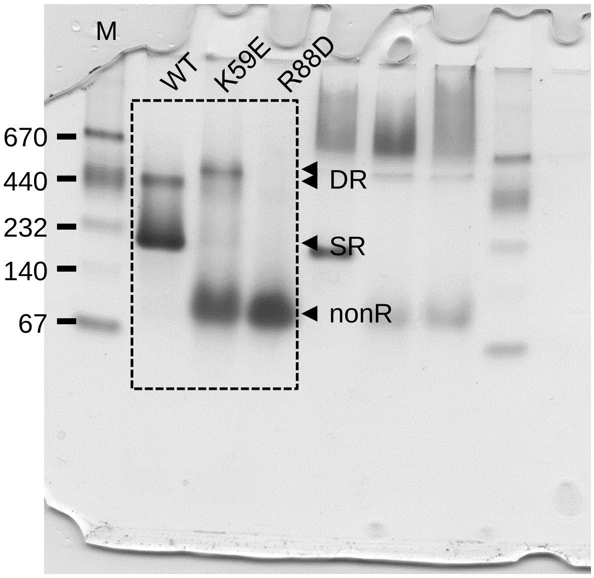

Figure 2b

These are the three panels of the composite sizing column figure for the K59E mutant.
M = molecular size standards (kDa).

left panel

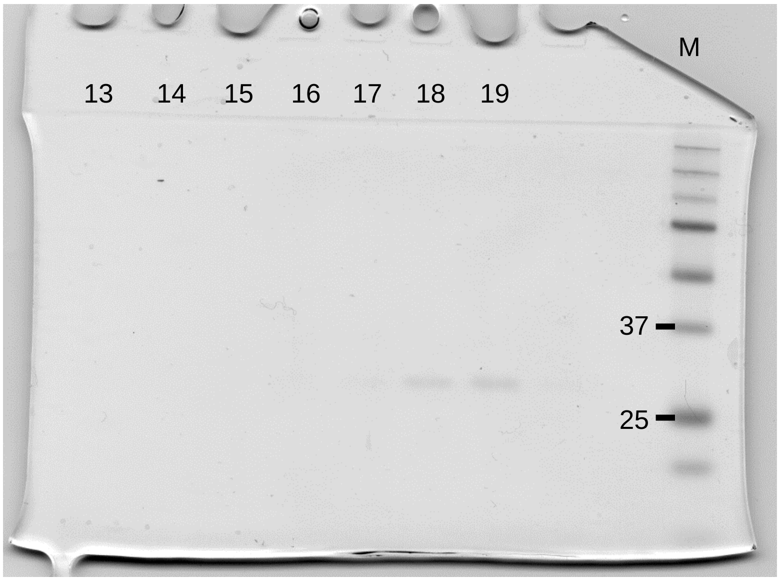

middle panel

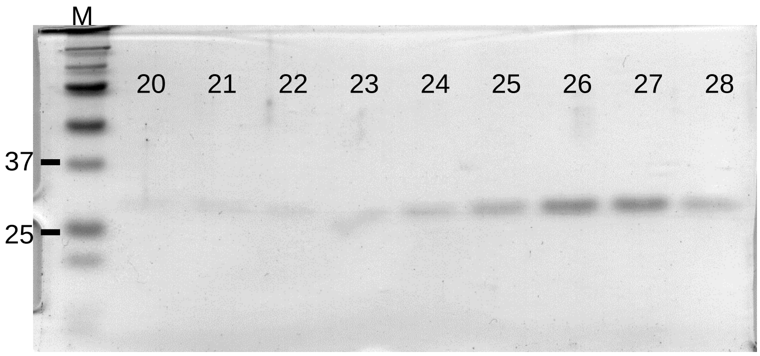

right panel

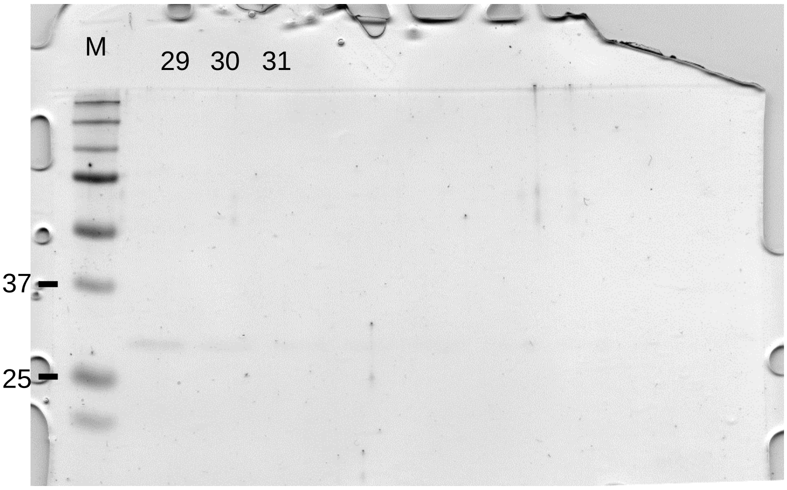

Figure 2c

These are the three panels of the composite sizing column figure for the R88D mutant.

M = molecular size standards (kDa).

left panel

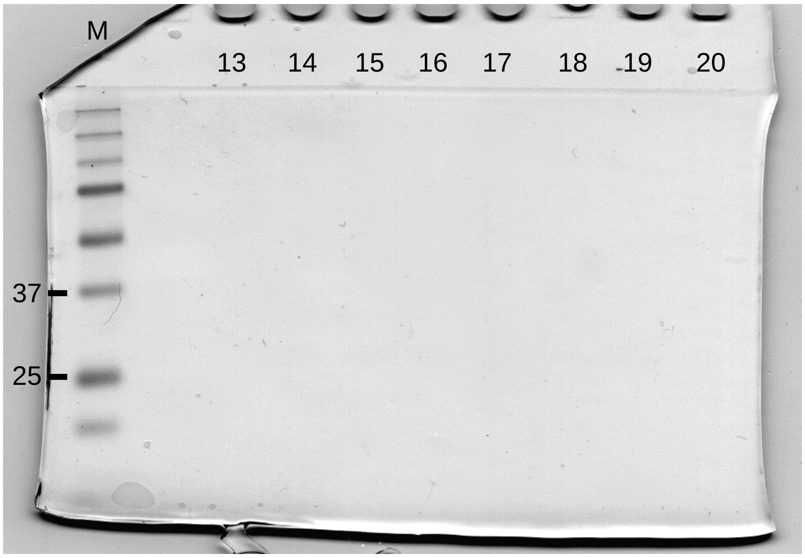

middle panel

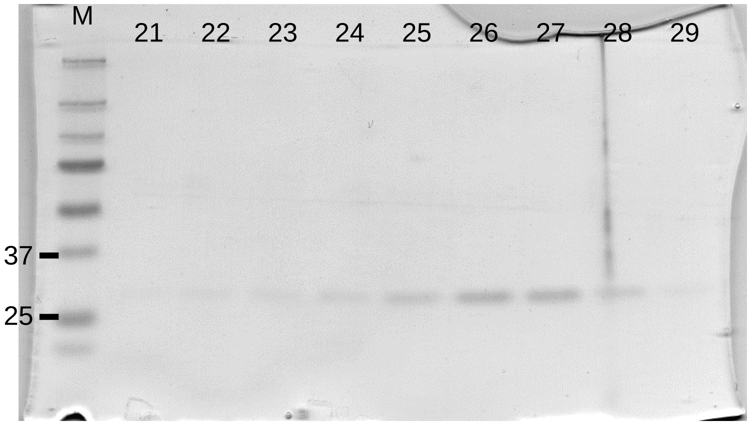

right panel

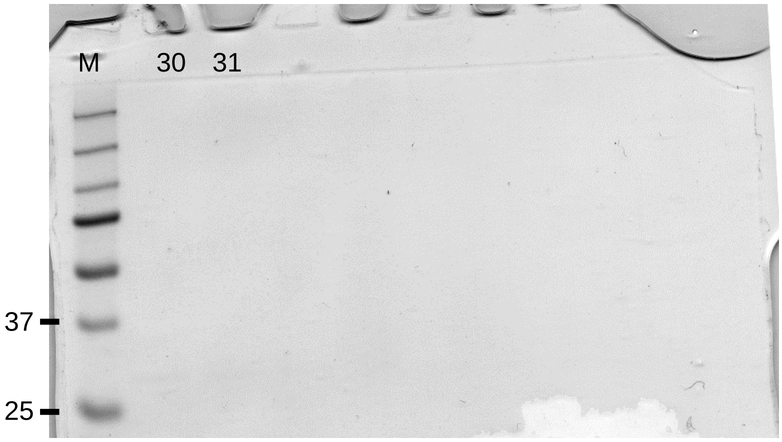

Figure 3
Dashed lines represent what is shown in the actual figure. M = molecular size standards (kDa)

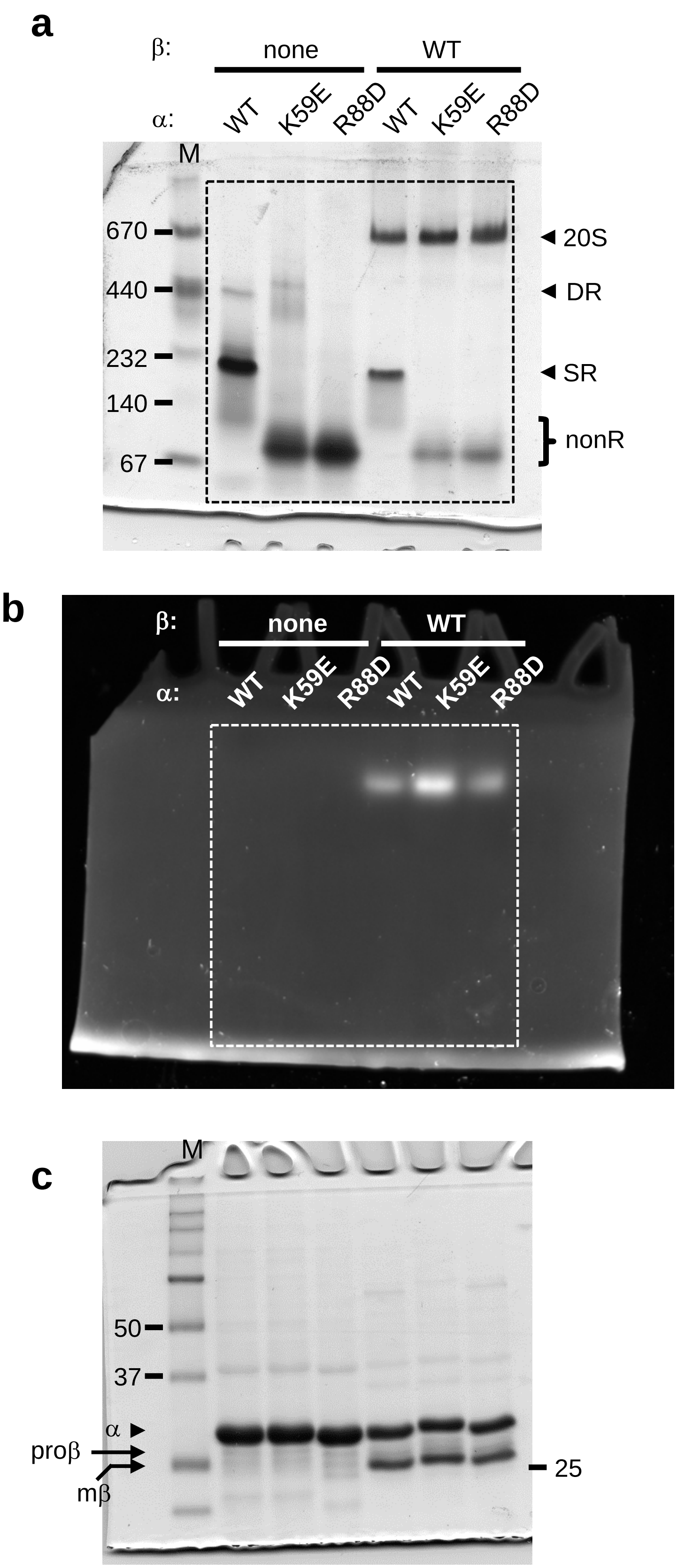

Figure 4
M = molecular size standards (kDa)

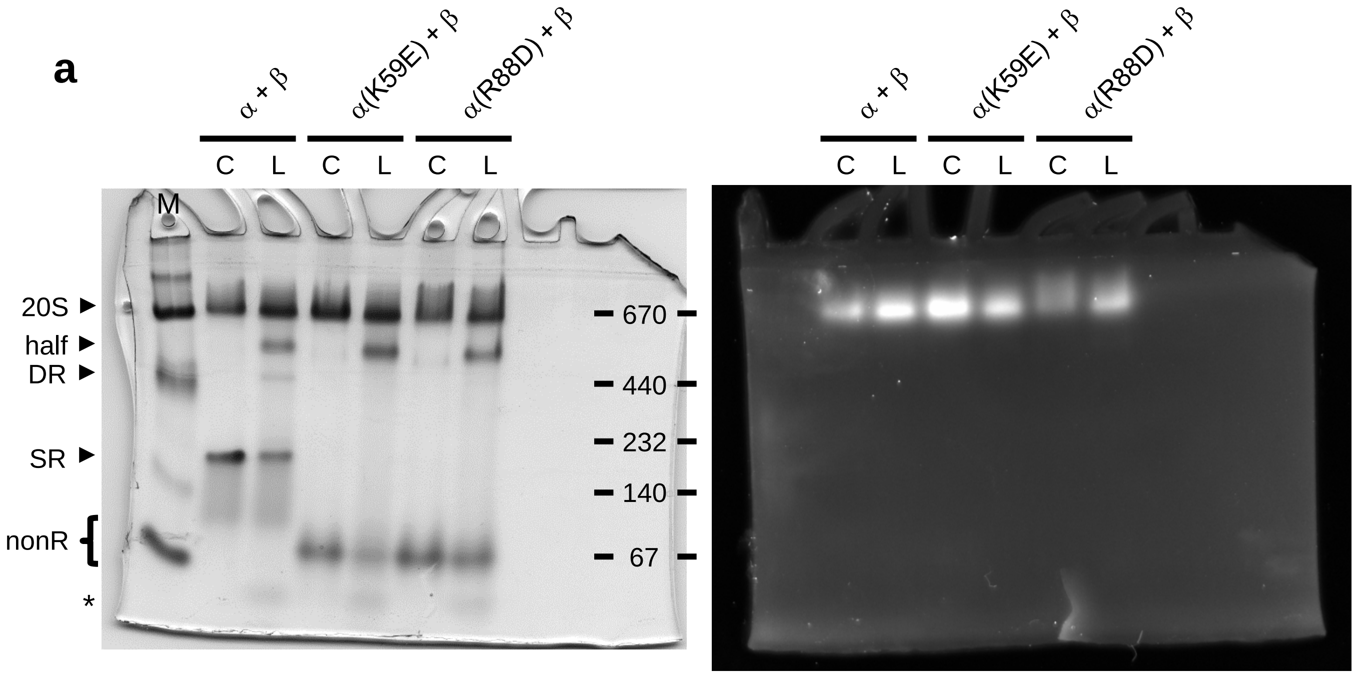

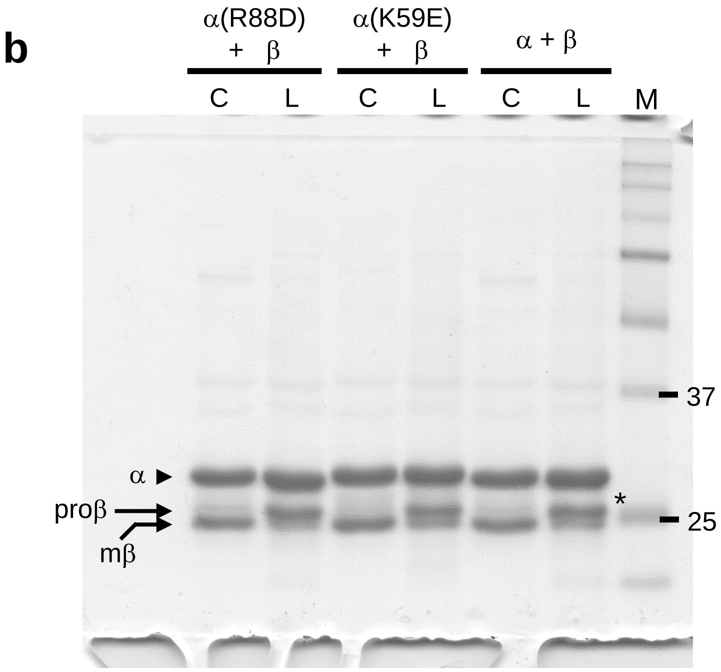

Figure 5
M = molecular size standards (kDa)

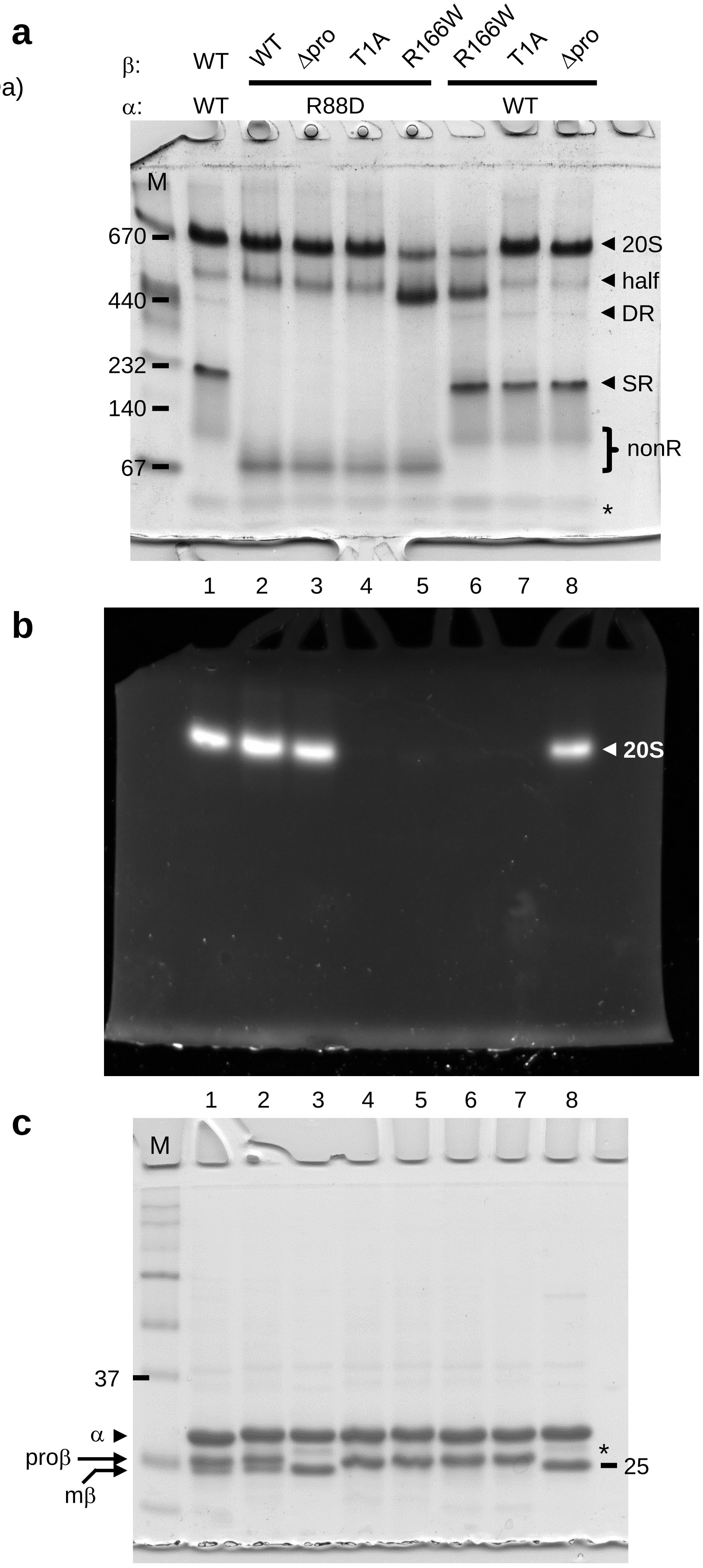

Figure 5 (continued)
M = molecular size standards (kDa)

d

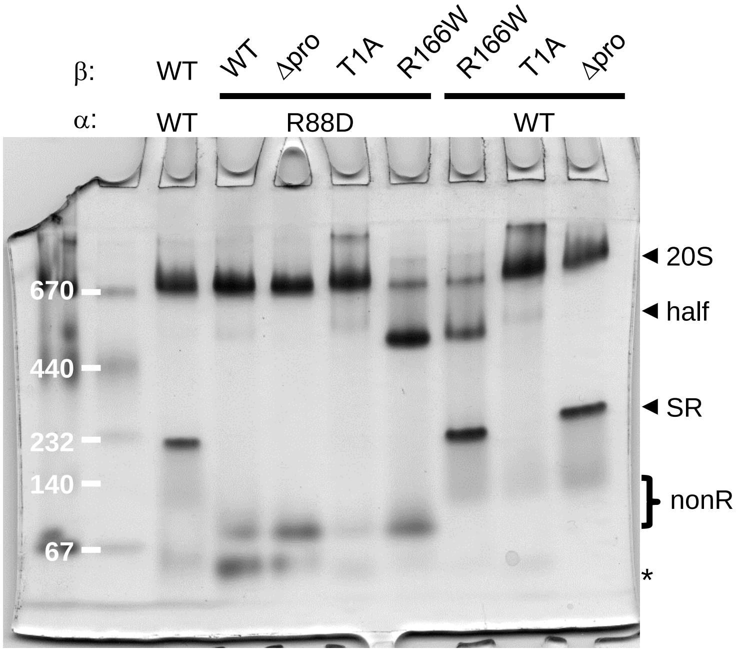

e

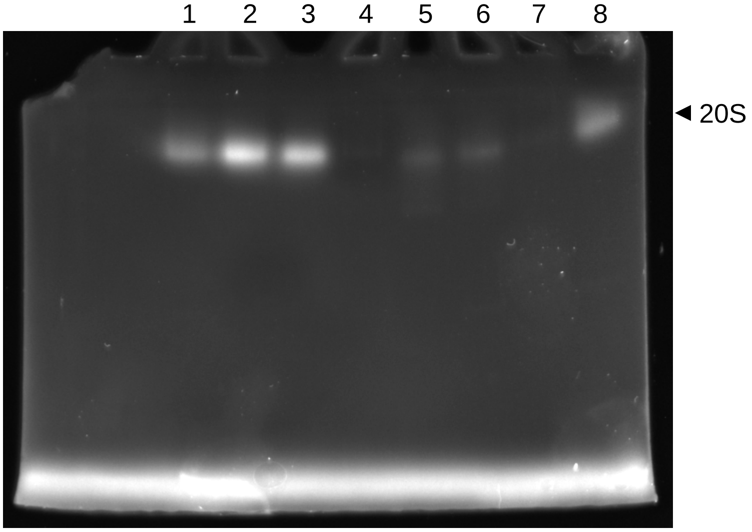

f

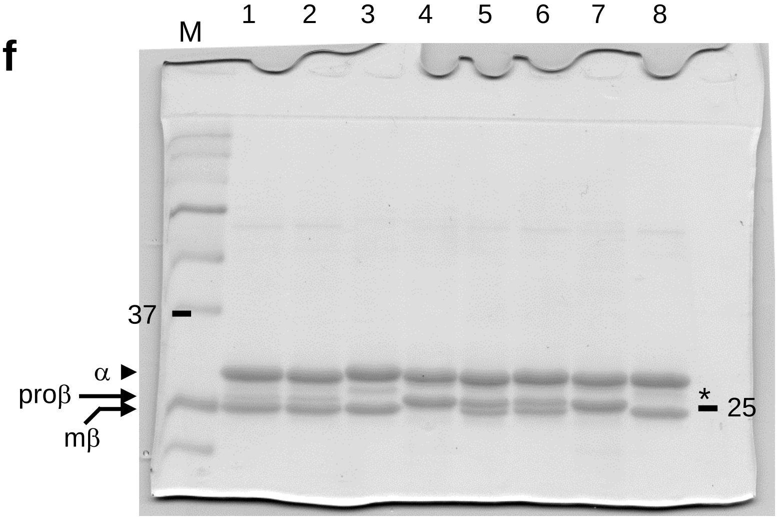

Figure 6
M = molecular size standards (kDa)

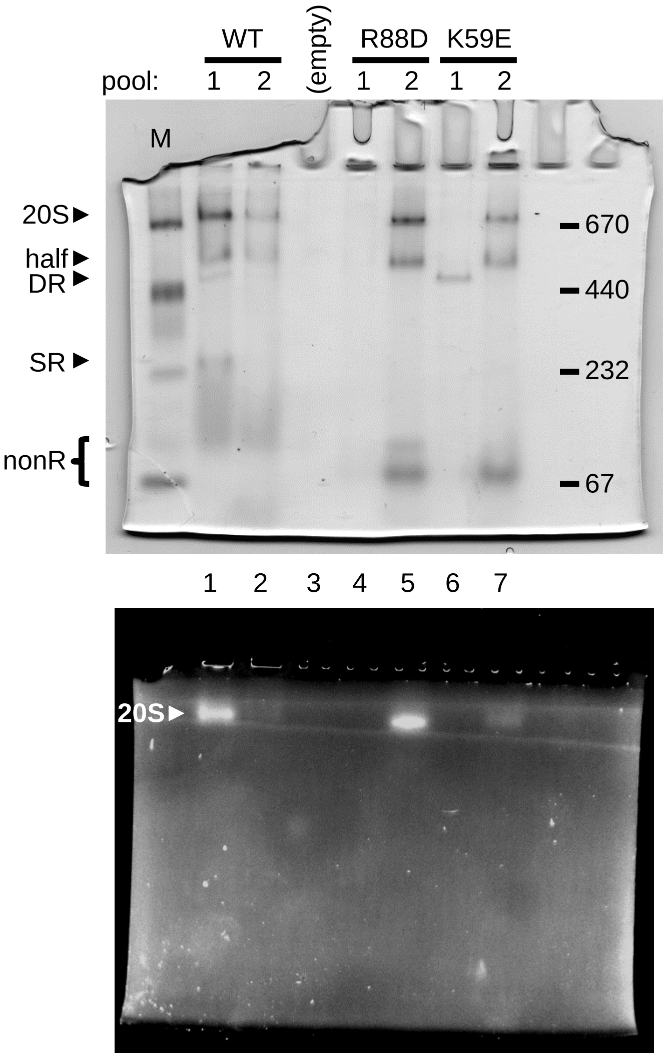

Supplement: Supplementary Full Gels [file srep13130-s2.pdf]
